# Gestational diabetes and ultrasound-assessed fetal growth in South Asian and White European women: findings from a prospective pregnancy cohort

**DOI:** 10.1186/s12916-018-1191-7

**Published:** 2018-11-06

**Authors:** Judith S. Brand, Jane West, Derek Tuffnell, Philippa K. Bird, John Wright, Kate Tilling, Debbie A. Lawlor

**Affiliations:** 1 MRC Integrative Epidemiology Unit, University of Bristol, Oakfield House, Oakfield Grove, Bristol, BS8 2BN UK; 2 Population Health Science, Bristol Medical School, Bristol, UK; 3 Clinical Epidemiology and Biostatistics, School of Medical Sciences, Örebro University, Örebro, Sweden; 4 Bradford Institute for Health Research, Bradford Royal Infirmary, Bradford, UK; 5 NIHR Bristol Biomedical Research Centre, Bristol, UK

**Keywords:** Gestational diabetes, Fetal growth, Ethnicity, Longitudinal trajectory analysis

## Abstract

**Background:**

Maternal gestational diabetes (GDM) is an established risk factor for large size at birth, but its influence on intrauterine fetal growth in different ethnic populations is less well understood. Here, we examine the joint associations of GDM and ethnicity with longitudinal fetal growth in South Asian and White European origin women.

**Methods:**

This study included 10,705 singletons (4747 White European and 5958 South Asian) from a prospective cohort of women attending an antenatal clinic in Bradford, in the North of England. All women completed a 75-g oral glucose tolerance test at 26–28 weeks’ gestation. Ultrasound measurements of fetal head circumference (HC), femur length (FL) abdominal circumference (AC), and estimated fetal weight (EFW), and corresponding anthropometric measurements at birth were used to derive fetal growth trajectories. Associations of GDM and ethnicity with these trajectories were assessed using multilevel fractional polynomial models.

**Results:**

Eight hundred thirty-two pregnancies (7.8%) were affected by GDM: 10.4% of South Asians and 4.4% of White Europeans. GDM was associated with a smaller fetal size in early pregnancy [differences (95% CI) in mean HC at 12 weeks and mean AC and EFW at 16 weeks comparing fetuses exposed to GDM to fetuses unexposed (reference) = − 1.8 mm (− 2.6; − 1.0), − 1.7 mm (− 2.5; − 0.9), and − 6 g (− 10; − 2)] and a greater fetal size from 24 weeks’ gestation through to term [differences (95% CI) in mean HC, AC, and EFW comparing fetuses exposed to GDM to those unexposed = 0.9 mm (0.3; 1.4), 0.9 mm (0.2; 1.7), and 7 g (0; 13) at 24 weeks]. Associations of GDM with fetal growth were of similar magnitude in both ethnic groups. Growth trajectories, however, differed by ethnicity with South Asians being smaller than White Europeans irrespective of GDM status. Consequently, South Asian fetuses exposed to GDM were smaller across gestation than fetuses of White Europeans without GDM.

**Conclusions:**

In both ethnic groups, GDM is associated with early fetal size deviations prior to GDM diagnosis, highlighting the need for novel strategies to diagnose pregnancy hyperglycemia earlier than current methods. Our findings also suggest that ethnic-specific fetal growth criteria are important in identifying hyperglycemia-associated pathological effects.

**Electronic supplementary material:**

The online version of this article (10.1186/s12916-018-1191-7) contains supplementary material, which is available to authorized users.

## Background

Gestational diabetes (GDM), which affects up to 10% of pregnancies depending on the population under study and diagnostic criteria applied, is associated with excess fetal growth resulting in large size at birth, adverse perinatal outcomes, and offspring adiposity [1]. Consistent evidence shows that despite having a greater risk of developing GDM, South Asian women give birth to lower weight infants than women of White European origin [2, 3]. This ethnic difference in weight appears to be present in the fetus from as early as 20 weeks’ gestation based on fetal ultrasound scan assessment [4–6].

Emerging evidence suggests that fetuses whose mothers go on to be diagnosed with GDM may already show accelerated growth before this diagnosis is made. Diagnostic testing, usually with an oral glucose tolerance test (OGTT), is undertaken at 26–28 weeks, because this is the time in pregnancy when there are detectable increases in insulin resistance [7]. In a study of largely White European women, faster hyperglycemia-related fetal growth was observed between 20 and 28 weeks, i.e., prior to the diagnostic test [8]. As there are effective and safe treatments for GDM that reduce birth size and perinatal morbidity [7, 9, 10], this finding suggests that earlier diagnosis might have clinical benefit. Whether a similar pattern of early growth deviation by GDM status would be seen in South Asian women who differ from White European women in fetal growth characteristics and risk of developing GDM is unknown. Moreover, as GDM is the upper end of a continuum of linear associations of increasing gestational glucose with greater birth weight [11–13] it is plausible that glucose levels below the diagnostic threshold will be related to fetal growth. Understanding the joint associations of GDM and South Asian ethnicity, two key risk factors for variation in birthweight, on fetal growth is important for identifying mechanisms that might explain lower birthweight in South Asians despite their greater GDM risk, and also for determining whether hyperglycemia-related pathological growth in South Asians can be determined from fetal growth patterns.

The aim of this study was to determine the associations of GDM and gestational glucose with longitudinal fetal growth across different parameters [head circumference (HC), abdominal circumference (AC), femur length (FL), and estimated fetal weight (EFW)] in White European and South Asian women.

## Methods

### Participants

We used data from the Born in Bradford (BiB) study, a population-based prospective pregnancy cohort including 12,450 women who experienced 13,773 pregnancies [14]. The cohort is broadly representative of the obstetric population in Bradford, a city in the north of England (UK), in which approximately half of the births are to mothers of South Asian origin. Eligible women had to have an expected delivery between March 2007 and December 2010 in the maternity department at Bradford Royal Infirmary (BRI). Participants were recruited primarily at their oral glucose tolerance test (OGTT) appointment, which is offered to all women booked for delivery at the BRI. Universal OGTT diagnosis of GDM has been implemented at the BRI since 2007 following a report by the Bradford Mortality Commission highlighting the rising infant mortality and poor health of pregnant women in Bradford [15]. Women who agreed to participate in BiB completed an interviewer-administered questionnaire, had their height and weight measured, and consented to the abstraction of medical records and fetal ultrasonography data. Ethical approval for the study was granted by the Bradford National Health Service Research Ethics Committee (ref 06/Q1202/48), and all participants gave written informed consent.

Figure S1 in Additional file 1 shows the flow of participants through the study. For this analysis, women had to have a singleton pregnancy without known congenital anomalies and with no history of pre-existing diabetes. We further restricted the cohort to women of White European and South Asian origin, as numbers from other ethnic backgrounds were too small to be analyzed separately. Following these exclusions, there were 11,697 eligible singletons, and of these 10,705 had data on GDM and fetal ultrasound.

### Assessment of fetal growth

Fetal ultrasound and birth anthropometric measurements were collected as part of the NHS screening program. In accordance with the National Institute for Health and Care Excellence (NICE) guidelines for UK antenatal care [16], women are invited to two routine ultrasound examinations during the first and second trimester: a “dating” scan performed between 10 weeks and 0 days and 13 weeks and 6 days’ gestation (but earlier and later gestational age assessment is common dependent on when the pregnant woman first presents to health services) and an “anomaly” scan which is offered between 18 weeks and 0 days and 20 weeks and 6 days’ gestation. In the UK, third trimester scans are not offered on a routine basis, but women deemed at a higher risk of pregnancy complications are offered additional scans, as required in the third trimester. In addition to these routine scans, a random sample of 3749 BiB participants were invited for a third trimester scan (at 32–34 weeks) as part of a sub-study investigating renal development [1806 (48%) completed the scan and of these 1569 met our inclusion criteria and are part of this study].

Based on UK ultrasonography recommendations [17], gestational age was determined using crown-rump length (CRL) up to 13 weeks 6 days, and head circumference thereafter. Measurements of HC, FL, and AC were done using standard ultrasound planes, and estimated fetal weight (EFW) was derived using the Hadlock 1985 formula [18], which has been validated in Europeans and South Asians [19]:

$$ {\mathrm{Log}}_{10}\ \mathrm{weight}=1.326-0.00326\ \mathrm{AC}\times \mathrm{FL}+0.0107\ \mathrm{HC}+0.0438\ \mathrm{AC}+0.158\ \mathrm{FL} $$

Infant HC, AC, and weight were measured within 24 h following birth by a pediatrician or specially trained midwife [20]. For the assessment of small (SGA) and large for gestational age (LGA), we used non-customized birth weight centiles (standardized by gestational age and sex) according to Intergrowth-21st standards [21]. We additionally defined SGA and LGA based on GROW customized centile charts using the UK bulk calculator from the Perinatal Institute [22]. As well as standardizing on gestational age and sex, these centiles also standardize on maternal parity, weight, height, and ethnicity, assuming these characteristics to be physiological, rather than pathological causes of SGA or LGA. In both methods, SGA and LGA were defined by the 10th and 90th percentiles, respectively, in infants delivered between 24 and 42 weeks’ gestation. Some of the additional data required for customization was missing and SGA and LGA based on customized charts were calculated for the 8318 with complete data on all parameters. To enable comparisons between those with and without complete data, we present results using the Intergrowth-21st standards for women with full data and also for the group in whom we were able to calculate SGA and LGA using customized charts (*n* = 8313).

### Maternal gestational diabetes and glucose levels

All women were offered a 75 g OGTT comprising overnight fasting and 2-h postload samples, at around 26–28 weeks [14]. Plasma glucose levels were assayed immediately after sampling using the glucose oxidase method on Siemen’s Advia 2400 chemistry autoanalyzers and Siemen’s Advia Centaur assay. Coefficients of variation range between 1.73% at 3.2 mmol/L and 0.64% at 19.1 mmol/L. GDM was defined according to modified WHO criteria operating at the time of the study as either fasting glucose ≥ 6.1 mmol/l or 2-h postload glucose ≥ 7.8 mmol/l [23].

### Ethnicity

Maternal ethnicity was determined at recruitment using interviewer-administered questionnaires, which asked participants to indicate which of the UK Office of National Statistics ethnicity categories best described their ethnic origin. Data on women’s report of country of their, their partners, and all four grandparents’ country of birth were used to verify their self-reported ethnic origin. For women who did not have ethnicity data collected at the recruitment interview, data were abstracted from primary care medical records, which use a similar categorization. Women classified as South Asian included those who indicated they were Pakistani, Indian, or Bangladeshi, and women classified as White European included those who indicated that they were White British or other White European origin.

### Covariates

Women were weighed and had their height measured (unshod and in light clothing) at recruitment (26–28 weeks’ gestation). Weight at the first antenatal clinic assessment when women had a median gestational age of 12 weeks [interquartile range (IQR) 11–14] was abstracted from the antenatal records, and this weight together with the measured height at recruitment, was used to calculate the woman’s early pregnancy BMI. Data on infant sex, parity, hypertensive disorders of pregnancy, and still births were abstracted from medical records. Information on maternal age at delivery, education, smoking, and alcohol consumption was derived from the interviewer-administered questionnaire at recruitment.

### Statistical analyses

Analyses were undertaken using MLwiN version 2.4 run in Stata version 15 [24]. Fetal growth trajectories were estimated using 2-degree fractional polynomial models in a multilevel framework [measurements within occasions (level 1) within individuals (level 2)]. Full specification of the models can be found in the Supplementary Methods in Additional file 2, including supportive data (Table S1 and Table S2 in Additional file 1) for the growth trajectories fitted (Figure S2 in Additional file 1).

Differences in fetal growth by ethnicity and GDM/gestational glucose were analyzed by adding these variables, and their interactions with gestational age to the multilevel models. Associations with gestational glucose in those without GDM were assessed by analyzing fasting and 2-h postload glucose levels as quintiles and per standard deviation (SD) increase. Differences in fetal size were estimated at 4-weekly intervals from 12/16 to 40 weeks’ gestation and are reported in absolute original units (i.e., millimeters and grams) and proportionally as the ratio of the observed difference to the mean at each time point. Results are presented with adjustment for infant sex (model 1) and with further adjustment for maternal age at delivery, parity, height, BMI, education level, smoking and alcohol use during pregnancy, and hypertensive disorders of pregnancy (model 2). Ethnic differences in associations between GDM/gestational glucose and fetal growth were assessed by adding relevant interaction terms to the models. In analyses of joint associations of ethnicity and GDM, we compared White European women with GDM, South Asian women without GDM and South Asian women with GDM, to White European women without GDM (the reference group).

Missing covariate data were imputed using multiple imputation (see details in the supplementary methods in Additional file 2), enabling a sample size of *n* = 10,700, 10,689, 10,520, and 10,701 for HC, FL, AC, and EFW respectively. For comparison, we also ran all analyses using complete case data (*n* = 8347, 8340, 8215, and 8349 for HC, FL, AC, and EFW respectively). To assess the possible influence of large numbers of repeat measurements, we did a sensitivity analysis limited to singletons with ≤ 4 ultrasound scans. We also evaluated the potential influence of OGTT timing in an analysis excluding pregnancies with an early (< 24 weeks) or late OGTT (> 30 weeks).

## Results

Maternal and infant characteristics of participants excluded from analyses because of missing data on GDM and/or ultrasound scan measures, were similar to those included in our study (Table S3 in Additional file 1). Characteristics of the analysis cohort by ethnicity and GDM status are summarized in Table 1. In total, 832 pregnancies were affected by GDM (7.8%). The prevalence of GDM was higher in South Asians (10.4%) than in White Europeans (4.4%), as were mean fasting and 2-h postload glucose levels (in those without GDM). Compared to White Europeans, South Asian women were shorter, had a lower BMI, higher parity, and were considerably less likely to drink alcohol or smoke during pregnancy. South Asian infants were also lighter at birth and less likely to be LGA by Intergrowth-21st standards. With GROW customized centile charts, the higher level of LGA in White Europeans decreased such that levels were similar to those in South Asians (as we might expect given these charts remove ethnic differences by assuming them to be physiological). Mean (SD) gestational age at OGTT was 26 (2.0) weeks. In both ethnic groups, women diagnosed with GDM were more likely to have a higher BMI and shorter stature than normoglycemic women, and infants of GDM pregnancies had a higher birthweight with a larger proportion being LGA.

**Table 1:** Characteristics of the singleton cohort by ethnicity and gestational diabetes status

	White European			South Asian		
	All (*N* = 4747)	No GDM (*N* = 4537)	GDM (*N* = 210)	All (*N* = 5958)	No GDM (*N* = 5336)	GDM (*N* = 622)
Infant characteristics						
Sex, % (*N*)						
Male	51.7 (2455)	51.8 (2351)	49.5 (104)	51.5 (3069)	51.4 (2743)	52.4 (326)
Female	48.3 (2292)	48.2 (2186)	50.5 (106)	48.5 (2889)	48.6 (2593)	47.6 (296)
Ethnicity by country, % (*N*)						
White British	92.6 (4396)	92.7 (4204)	91.4 (192)	–	–	–
White other	7.4 (351)	7.3 (333)	8.6 (18)	–	–	–
Pakistani	–	–	–	87.0 (5181)	87.1 (4649)	85.5 (532)
Indian	–	–	–	7.2 (43)	7.4 (392)	6.4 (40)
Bangladeshi	–	–	–	5.8 (345)	5.5 (295)	8.0 (50)
Birthweight (g), mean (SD)	3354 (550)	3359 (551)	3246 (450)	3140 (515)	3145 (517)	3094 (489)
Missing, % (*N*)	0.2 (8)	0.2 (8)	0 (0)	0.03 (2)	0.04 (2)	0 (0)
Gestational age at birth (years), mean (SD)	39.3 (1.8)	39.4 (1.8)	38.1 (1.5)	39.1 (1.7)	39.2 (1.7)	38.2 (1.3)
Missing, % (*N*)	0.2 (8)	0.2 (8)	0 (0)	0.03 (2)	0.04 (2)	0 (0)
Intergrowth birth weight centiles (full cohort analyses), % (*N*)*						
SGA (< 10th)	8.0 (377)	8.1 (368)	4.3 (9)	16.2 (962)	16.7 (891)	11.4 (71)
LGA (> 90th)	14.9 (707)	14.8 (669)	18.1 (38)	7.0 (419)	6.6 (354)	10.5 (65)
Missing	0.2 (8)	0.2 (8)	0 (0)	0.03 (2)	0.04 (2)	0 (0)
Intergrowth birth weight centiles (restricted analyses), % (*N*)*						
SGA (< 10th)	7.1 (275)	7.3 (268)	3.6 (7)	16.4 (730)	17.0 (677)	11.3 (53)
LGA (> 90th)	15.1 (584)	15.0 (552)	16.8 (32)	6.7 (297)	6.2 (246)	10.8 (51)
Missing	18.6 (884)	19.0 (864)	9.5 (20)	25.3 (1508)	25.4 (1357)	24.3 (151)
Customized birth weight centiles, % (*N*)**						
SGA (< 10th)	16.4 (634)	16.7 (614)	10.5 (20)	15.0 (670)	15.5 (617)	11.3 (53)
LGA (> 90th)	4.8 (186)	4.6 (170)	8.4 (16)	6.0 (269)	5.5 (220)	10.4 (49)
Missing	18.6 (884)	19.0 (864)	9.5 (20)	25.3 (1508)	25.4 (1357)	24.3 (151)
Maternal characteristics						
Age at delivery (years), mean (SD)	26.7 (6.0)	26.6 (6.0)	30.2 (5.4)	28.0 (5.1)	27.7 (5.0)	30.7 (5.3)
Parity, % (*N*)						
Primiparous	48.5 (2238)	48.6 (2144)	46.1 (94)	32.1 (1843)	32.9 (1692)	25.0 (151)
1	30.6 (1413)	30.6 (1349)	31.4 (64)	27.5 (1577)	28.3 (1455)	20.2 (122)
2	13.1 (603)	13.0 (573)	14.7 (30)	20.5 (1180)	20.3 (1045)	22.4 (135)
≥ 3	7.8 (358)	7.8 (342)	7.8 (16)	19.9 (1145)	18.5 (949)	32.5 (196)
Missing	2.8 (135)	2.8 (129)	2.9 (6)	3.6 (213)	3.7 (195)	2.9 (18)
Height (cm), mean (SD)	164.1 (6.2)	164.2 (6.3)	163.8 (6.1)	159.4 (5.8)	159.6 (5.8)	157.9 (5.8)
Missing, % (*N*)	12.6 (598)	12.9 (587)	5.2 (11)	18.3 (1090)	18.4 (981)	17.5 (109)
Body mass index (kg/m^2^), mean (SD)	26.6 (6.0)	26.5 (5.9)	28.6 (6.3)	25.5 (5.4)	25.2 (5.3)	28.2 (5.8)
Missing, % (*N*)	14.5 (689)	14.9 (674)	7.1 (15)	19.6 (1166)	19.6 (1046)	19.3 (120)
Education, % (*N*)						
< 5 GCSEs	19.4 (799)	19.6 (770)	14.7 (29)	24.7 (1208)	23.9 (1045)	31.5 (163)
5 GCSEs	34.1 (1404)	34.4 (1350)	27.4 (54)	30.9 (1513)	30.9 (1353)	31.0 (160)
A-level	17.3 (714)	17.4 (681)	16.8 (33)	12.8 (625)	13.3 (583)	8.1 (42)
Higher than A-level	20.8 (857)	20.3 (796)	31.0 (61)	28.4 (1389)	28.7 (1255)	25.9 (134)
Other	8.3 (343)	8.2 (323)	10.2 (20)	3.3 (163)	3.3 (145)	3.5 (18)
Missing	13.3 (630)	13.6 (617)	6.2 (13)	17.8 (1060)	17.9 (955)	16.9 (105)
Maternal characteristics						
Smoking during pregnancy, % (*N*)						
No	67.4 (2836)	67.0 (2682)	76.2 (154)	96.8 (4817)	96.8 (4307)	96.6 (510)
Yes	32.6 (1371)	33.0 (1323)	23.8 (48)	3.2 (159)	3.2 (141)	3.4 (18)
Missing	11.4 (540)	11.7 (532)	3.8 (8)	16.5 (982)	16.6 (888)	15.1 (94)
Any alcohol during pregnancy, % (*N*)						
No	33.3 (1399)	33.1 (1323)	37.6 (76)	98.7 (4908)	98.7 (4389)	98.5 (519)
Yes	66.7 (2802)	66.9 (2676)	62.4 (126)	1.4 (67)	1.3 (59)	1.5 (8)
Missing	11.5 (546)	11.9 (538)	3.8 (8)	16.5 (983)	16.6 (888)	15.3 (95)
Fasting glucose (mmol/L), mean (SD)	4.40 (0.41)	4.39 (0.37)	4.86 (0.82)	4.63 (0.61)	4.53 (0.42)	5.42 (1.17)
Missing	10.0 (475)	10.3 (468)	3.3 (7)	8.0 (479)	8.1 (448)	5.0 (31)
2-h postload glucose (mmol/L), mean (SD)	5.44 (1.28)	5.28 (1.07)	8.55 (1.20)	5.88 (1.64)	5.48 (1.02)	9.13 (2.13)
Missing	10.1 (481)	10.4 (474)	3.3 (7)	8.2 (486)	8.5 (455)	5.0 (31)
Hypertensive disorders of pregnancy, % (*N*)						
No	93.3 (4427)	93.3 (4231)	93.3 (196)	94.7 (5644)	94.8 (5057)	94.4 (587)
Yes	6.7 (320)	6.7 (306)	6.7 (14)	5.3 (314)	5.2 (279)	5.6 (35)

*GDM* gestational diabetes, *SD* standard deviation, *GCSE* General Certificate of Secondary Education. *SGA- and LGA-defined based on non-customized birth weight centiles (standardized on sex and gestational age only) according to Intergrowth-21st standards. **SGA- and LGA-defined based on GROW customized birth weight centiles (standardized on sex, gestational age, maternal parity, height, and weight). For the Intergrowth-21st (non-customized) charts, we present results for the maximal sample (*n* = 10,695) and for the smaller sample (*n* = 8313 with no missing data on maternal parity, height, and weight) used for comparison with customized birth weight centiles. Please note that the Intergrowth-21st standards and GROW customized birth weight centiles use different methods, with different principles regarding health birth size. Thus, even where two proportions are similar, it is likely that different participants contribute to the cases of SGA and LGA

Table S2 in Additional file 1 summarizes the repeat anthropometric measurements included in the analyses. The median number of anthropometric measures per fetus was 3 (IQR 2–4; full-range 1–12) and the mean (SD) gestational age at first fetal anthropometric measurement was 18.8 (3.1) weeks. As might be expected, women with a third trimester scan were older, had a higher BMI, were more often multiparous, and were more likely to have diagnosis of GDM or hypertensive disorder of pregnancy (Table S4 in Additional file 1**)**. Infants of women with a third trimester scan were delivered with an earlier gestational age and had a lower birthweight than infants of mothers who did not have a third trimester scan (Table S4 in Additional file 1**)**. In total, 2341 participants did not have a CRL measurement for dating and fetal HC was used to date these women. Infants of the 2341 women who were “dated” on the basis of HC rather than CRL had similar gestational ages at delivery and birthweights; their mothers had similar mean BMI, height, fasting and postload glucose, and rates of GDM and hypertensive disorder of pregnancy. They were, however, more likely to be South Asian, have a higher parity, lower education, and less likely to smoke or drink alcohol in pregnancy (Table S5 in Additional file 1**)**.

### Ethnicity and fetal growth

Ethnic differences in fetal growth trajectories of HC, FL, AC, and EFW are presented in Figure S3 in Additional file 1. Except for FL, South Asian fetuses were smaller than White European fetuses from 20 weeks’ gestation through to birth, with the largest difference observed for AC and EFW (Table 2 and Figure S3 in Additional file 1). Absolute and proportional differences in fetal size increased with increasing gestational age, and the difference in means (95% CI) comparing South Asians to White Europeans for AC and EFW were − 3.3 mm (− 4.0; − 2.5) and − 17 g (− 24; − 11) at 24 weeks, and − 12.4 mm (− 13.9; − 10.9) and − 206 g (− 232; − 180) at 40 weeks respectively. Mean differences in HC by ethnicity were smaller [− 1.0 mm (− 1.5; − 0.5) at 24 weeks and − 5.4 mm (− 6.1; − 4.6) at 40 weeks comparing South Asians to White Europeans]. There was also some evidence of South Asian fetuses having a greater FL between 20 and 28 weeks’ gestation but not towards the end of the third trimester.

**Table 2:** Predicted mean differences of fetal parameters by ethnicity and gestational diabetes at different time points during gestation

		Predicted mean difference (95% CI)		
HC (mm)	*N*	12 weeks	24 weeks	40 weeks
Ethnicity				
White European	4744	Ref	Ref	Ref
South Asian	5956	0.3 (− 0.5; 1.0)	− 1.0 (− 1.5; − 0.5)	− 5.4 (− 6.1; − 4.6)
GDM				
No	9868	Ref	Ref	Ref
Yes	832	− 1.8 (− 2.6; − 1.0)	0.9 (0.3; 1.4)	0.1 (− 0.8; 1.1)
Ethnicity/GDM				
White European/no GDM	4534	Ref	Ref	Ref
White European/GDM	210	− 2.7 (− 4.3; − 1.1)	1.5 (0.5; 2.6)	0.1 (− 1.7; 1.9)
South Asian/no GDM	5334	0.2 (− 0.6; 0.9)	− 0.9 (− 1.4; − 0.4)	− 5.3 (− 6.1; − 4.5)
South Asian/GDM	622	− 1.4 (− 2.5; − 0.3)	− 0.3 (− 1.0; 0.5)	− 5.2 (− 6.5; − 4.0)
FL (mm)	*N*	12 weeks	24 weeks	40 weeks
Ethnicity				
White European	4740	Ref	Ref	Ref
South Asian	5949	0.1 (− 0.2; 0.5)	0.3 (0.1; 0.4)	0.0 (− 0.4; 0.3)
GDM				
No	9857	Ref	Ref	Ref
Yes	832	− 0.6 (− 0.9; − 0.3)	0.1 (− 0.1; 0.2)	0.1 (− 0.2; 0.4)
Ethnicity/GDM				
White European/no GDM	4530	Ref	Ref	Ref
White European/GDM	210	0.1 (− 0.6; 0.7)	0.1 (− 0.2; 0.4)	0.6 (0.1; 1.1)
South Asian/no GDM	5327	0.2 (− 0.1; 0.6)	0.3 (0.1; 0.4)	0.1 (− 0.3; 0.4)
South Asian/GDM	622	− 0.6 (− 1.1; − 0.2)	0.3 (0.1; 0.5)	0.0 (− 0.5; 0.4)
AC (mm)	*N*	16 weeks	24 weeks	40 weeks
Ethnicity				
White European	4648	Ref	Ref	Ref
South Asian	5872	− 1.5 (− 2.3; − 0.8)	− 3.3 (− 4.0; − 2.5)	− 12.4 (− 13.9; − 10.9)
GDM				
No	9690	Ref	Ref	Ref
Yes	830	− 1.7 (− 2.5; − 0.9)	0.9 (0.2; 1.7)	5.1 (3.5; 6.8)
Ethnicity/GDM				
White European/no GDM	4438	Ref	Ref	Ref
White European/GDM	210	− 2.2 (− 3.7; − 0.7)	0.9 (− 0.5; 2.3)	4.3 (1.1; 7.5)
South Asian/no GDM	5252	− 1.6 (− 2.4; − 0.8)	− 3.2 (− 4.0; − 2.5)	− 12.5 (− 14.0; − 11.0)
South Asian/GDM	620	− 3.1 (− 4.3; − 2.0)	− 2.3 (− 3.4; − 1.2)	− 7.1 (− 9.4; − 4.8)
EFW (g)	*N*	16 weeks	24 weeks	40 weeks
Ethnicity				
White European	4745	Ref	Ref	Ref
South Asian	5956	2 (− 2; 6)	− 17 (− 24; − 11)	− 206 (− 232; − 180)
GDM				
No	9869	Ref	Ref	Ref
Yes	832	− 6 (− 10; − 2)	7 (0; 13)	171 (140; 202)
Ethnicity/GDM				
White European/no GDM	4535	Ref	Ref	Ref
White European/GDM	210	− 3 (− 11; 4)	5 (− 6; 17)	204 (145; 263)
South Asian/no GDM	5334	2 (− 2; 6)	− 17 (− 24; − 11)	− 203 (− 230; − 177)
South Asian/GDM	622	− 5 (− 11; 0)	− 10 (− 19; − 1)	− 45 (− 86; − 3)

Separate and joint associations of ethnicity and gestational diabetes with fetal growth trajectories, presented as mean differences of head circumference (HC), femur length (FL), abdominal circumference (AC), and estimated fetal weight (EFW) during early (12/16 weeks), mid (24 weeks), and late (40 weeks) gestation. All mean differences are estimated using multilevel fractional polynomial models with adjustment for infant sex, maternal age at delivery, parity, height, body mass index, education level, smoking, and alcohol use during pregnancy, and hypertensive disorders of pregnancy. Models were additionally adjusted for gestational diabetes [in analyses examining mean differences by ethnicity (White European vs. South Asian)] and ethnicity [in analyses examining mean differences by gestational diabetes (yes vs. no)]. *GDM* gestational diabetes, *CI* confidence interval

### GDM, gestational glucose, and fetal growth

Trajectories of individual fetal growth parameters by GDM status are presented in Figure S4 in Additional file 1. Fetuses of women who were later diagnosed with GDM were smaller in early pregnancy [difference in mean HC at 12 weeks and mean AC and EFW at 16 weeks comparing fetuses exposed to GDM to fetuses not exposed to GDM (reference) = − 1.8 mm (− 2.6; 1.0), − 1.7 mm (− 2.5; − 0.9), and − 6 g (− 10; − 2) respectively]. This pattern of early growth restriction was followed by enhanced growth with fetuses of GDM complicated pregnancies being larger at 24 weeks’ gestation [difference in means comparing fetuses exposed to GDM vs. those unexposed were 0.9 mm (0.3; 1.4) for HC, 0.9 mm (0.2; 1.7) for AC and 7 g (0; 13) for EFW respectively] (Table 2 and Figure S4 in Additional file 1). From 24 weeks to delivery, absolute and proportional differences in AC and EFW increased with increasing gestational age [mean differences associated with GDM at 40 weeks’ gestation were 5.1 mm (3.5–6.8) and 171 g (140; 202) respectively], while the larger HC observed at 24 weeks attenuated towards the end of pregnancy and was no longer detectable at term (mean difference = 0.1 mm (− 0.8; 1.1) at 40 weeks). The FL growth trajectory did not notably differ by GDM status across pregnancy.

To evaluate whether associations of GDM with fetal growth are continuous across the glucose distribution, we also assessed associations with gestational fasting and 2-h postload glucose levels in women who were not diagnosed with GDM. These results are presented in Table S6 in Additional file 1; Figure S5 and S6 in Additional file 1. Gestational glucose levels were positively and linearly associated with fetal AC and EFW starting from 20 to 24 weeks’ gestation and also with fetal HC from 28 weeks’ gestation. Overall, associations with HC, AC, and EFW were somewhat weaker for 2-h postload glucose than fasting glucose levels. While the mean difference in fetal HC observed with GDM did not persist to term, gestational glucose levels below the diagnostic threshold were positively associated with fetal HC until birth. As with GDM, there was no clear evidence of gestational glucose being associated with FL trajectories (Table S6 in Additional file 1; Figure S5 and S6 in Additional file 1).

### Joint associations of ethnicity and GDM with fetal growth

**Fig. 1:**
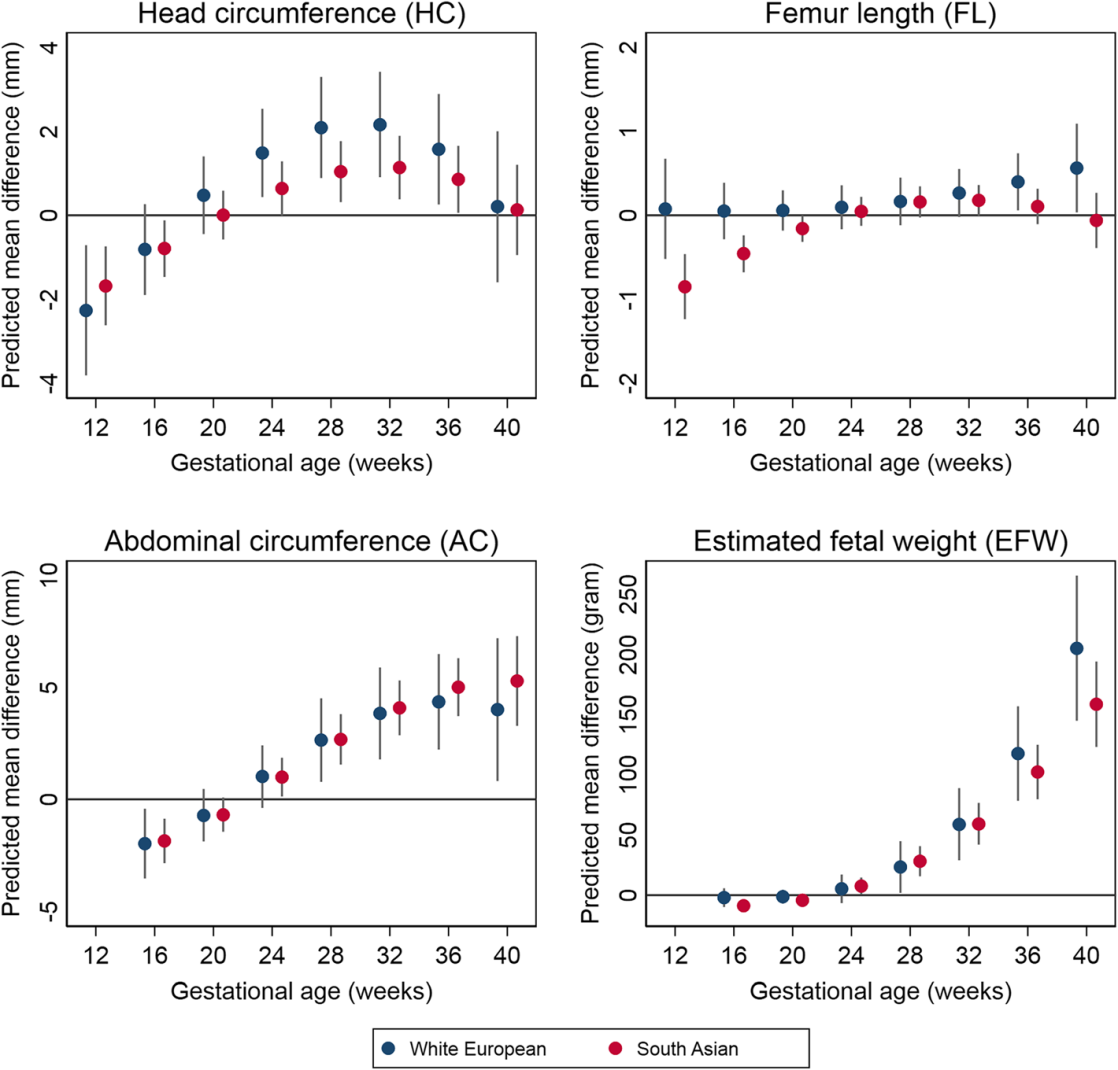
Associations of gestational diabetes with fetal growth across gestation in White Europeans and South Asians. Predicted differences in mean head circumference (mm), femur length (mm), abdominal circumference (mm), and estimated fetal weight (g) comparing fetuses exposed to gestational diabetes to those not exposed to gestational diabetes (reference group) across gestation. Predicted mean differences are given for the total study population and for each ethnic group (White European and South Asian). All estimates are derived from multivariable models with adjustment for infant sex, maternal age at delivery, ethnicity, parity, height, body mass index, education level, smoking, and alcohol use during pregnancy and hypertensive disorders of pregnancy. Positive mean differences (larger than 0) indicate larger size in fetuses exposed to gestational diabetes

Figure 1 shows the observed mean differences in fetal size across gestation by GDM in each ethnic group. There was no evidence of effect modification by ethnicity, as the direction and magnitude of associations of GDM with fetal growth were similar in South Asians and White Europeans, as were associations with postload glucose in those not diagnosed with GDM (Figure S5 in Additional file 1**)**. Positive associations of fasting glucose with fetal growth were somewhat stronger in White European than South Asian fetuses, but as with GDM, broadly similar in each ethnic group (Figure S6 in Additional file 1).

**Fig. 2:**
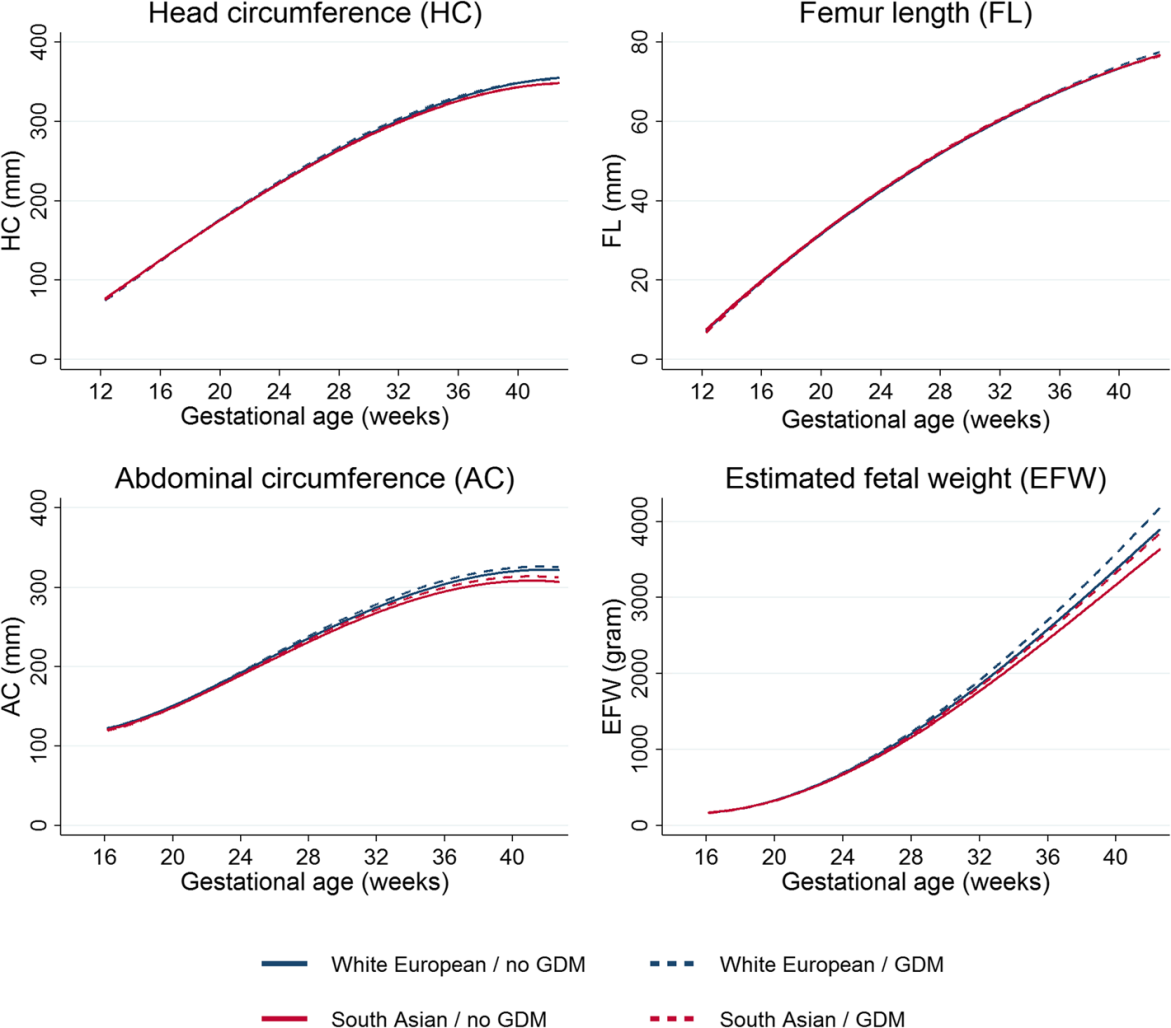
Joint associations of ethnicity and gestational diabetes with fetal growth. Average predicted growth trajectories of head circumference (*A*), femur length (*B*), abdominal circumference (*C*), and estimated fetal weight (*D*) stratified by ethnic origin (South Asian vs. White European) and gestational diabetes status (yes vs. no). All growth trajectories are estimated using multilevel fractional polynomial models with adjustment for infant sex, maternal age at delivery, parity, height, body mass index, education levels, smoking, and alcohol use during pregnancy, and hypertensive disorders of pregnancy.

As the association of GDM with fetal growth did not differ by ethnicity, and South Asians were consistently smaller in size than White Europeans across pregnancy for most growth measures (independently of GDM), South Asian fetuses exposed to GDM were smaller across gestation than White European fetuses not exposed to GDM **(**Fig. 2, Table 2). This difference was largest for AC and EFW and detectable from early pregnancy [difference in mean AC and EFW at 24 weeks comparing South Asians exposed to GDM to White Europeans not exposed to GDM = − 2.3 mm (− 3.4; − 1.2) and − 10 g (− 19; − 1) respectively] and increased as pregnancy progressed, such that by term (40 weeks) it was − 7.1 mm (− 9.4; − 4.8) for AC and − 45 g (− 86; − 4) for EFW.

All results presented above are from multivariable adjusted analyses following multiple imputation for missing covariable data; associations were similar in analyses with adjustment for infant sex only (Table S7 in Additional file 1) and in complete case analyses (Table S8 in Additional file 1). They were also similar when limited to women with ≤ 4 repeat ultrasound scans (Table S9 in Additional file 1) and when limited to women who completed the OGTT between 24 and 30 weeks’ gestation (Table S10 in Additional file 1).

## Discussion

In this prospective pregnancy cohort, we found that fetuses of women subsequently diagnosed with GDM were smaller at 12–16 weeks’ gestation but grew faster such that from 24 weeks to delivery time, they had greater AC and EFW compared with fetuses not exposed to GDM. The association of GDM with fetal growth was of similar magnitude in both ethnic groups and was also observed across the distribution of fasting and postload glucose levels in women without GDM. Growth trajectories differed by ethnicity irrespective of GDM status, with South Asian fetuses being smaller from 20 weeks’ gestation to term, than White European fetuses. Consequently, South Asian fetuses exposed to GDM were on average smaller across gestation than White European fetuses not exposed to GDM. These findings indicate that hyperglycemia-associated fetal growth deviations are detectable early in pregnancy prior to the usual time of GDM diagnostic testing and that universal criteria for fetal growth assessment may be inadequate for identifying GDM-associated pathological effects in South Asians.

The observation of South Asians having a smaller fetal size across gestation than White Europeans is consistent with observations in other population-based studies [4–6, 25]. Ethnic differences in fetal size increased as the pregnancy progressed and were relatively more profound for AC and EFW than for HC. It has been suggested that the slower fetal growth in South Asians represents compromised growth and development of abdominal visceral organs with relative sparing of the fetal brain to improve survival chances in environments where nutritional resources may be limited, with this being driven by fetal adaptations to generations of maternal undernutrition [6, 26]. The fact that we see larger ethnic differences for AC and EFW than HC provides some support for this hypothesized mechanism. South Asian infants are, however, more adipose at birth on an absolute scale and also relative to their birth weight compared to White European infants [27, 28]. This “thin-fat phenotype” has also been observed in South Asian infants born in the UK and compared with White European infants born in the UK (including from the same city and treated in the same antenatal care setting) [3, 29]. It appears to persist through infancy and early childhood [30]. Since our study was based on standard ultrasound anthropometric measurements, we were unable to distinguish fetal fat and lean mass growth trajectories.

While the impact of GDM on birthweight is well-established [12, 31], few studies have assessed the timing at which divergent growth patterns emerge in utero [8, 32, 33]. Our findings of a larger fetal AC at 24 weeks’ gestation in women later diagnosed with GDM is consistent with previous observations in White European women [8], and support the notion that glycemia-related fetal growth acceleration precedes the usual time of OGTT to diagnose GDM. For the first time, we demonstrate a similar pattern of increased fetal size preceding the GDM diagnosis in South Asian origin women. Consistent with previous data on offspring birth weight [11–13], associations with GDM were part of a continuum as gestational glucose levels were positively and linearly associated with fetal size from 24 weeks onwards. The continuous positive association of gestational glucose across the whole distribution with fetal growth and birth weight, including in two ethnic groups with very different body compositions, likely reflects the facilitated diffusion of glucose across the placenta and the fetal insulin secretion response to this. The diagnostic criteria for GDM aim to minimize LGA and associated adverse outcomes, while not compromising healthy fetal growth and development, and the associations of fasting and postload glucose with fetal growth and birth weight in women who do not meet criteria for a diagnosis of GDM do not imply the thresholds for diagnosing GDM should be reduced. Our results do support developing methods for identifying hyperglycemia-related adverse fetal growth in early pregnancy.

We further found evidence that fetuses of GDM complicated pregnancies were smaller at 12 and 16 weeks’ gestation prior to the onset of growth acceleration. This biphasic fetal growth pattern with early fetal growth restriction predating growth acceleration has previously been described in GDM [32], pre-gestational type 1 [34, 35], and type 2 diabetes [33] and has been attributed to transient oxygen tension in early pregnancy with oxidative and/or proinflammatory stress superimposed by hyperglycemia exerting a short-term inhibitory effect on trophoblast and placental growth [36]. In our cohort, there were too few women (67 in total; 36 South Asian, 24 White European, and 7 other ethnic origin) to be able to explore fetal growth trajectories by pre-gestational diabetes.

Oral glucose tolerance tests for diagnosis of GDM are undertaken at 26–28 weeks because this is the time in pregnancy when there are detectable increases in insulin resistance [7]. However, GDM is likely to reflect the unmasking of a predisposition to hyperglycemia and type 2 diabetes as a result of pregnancy changes mimicking a glucose stress test [37]. This would be in line with reports linking pregestational increased glucose levels [38] and reductions in peripheral insulin sensitivity [39, 40] to a GDM diagnosis later in pregnancy. The International Association of the Diabetes and Pregnancy Study Groups’ criteria recommend a random fasting glucose measure in the first antenatal clinic visit which could identify women with pre-existing hyperglycemia and support earlier monitoring and treatment. Given we have shown GDM-associated fetal growth alterations as early as 12–16 weeks’ gestation, and other studies show early growth retardation in relation to existing type 1 and type 2 diabetes [33–35], efforts to improve pre- and periconceptional maternal health deserve more attention in the prevention of adverse pregnancy outcomes including hyperglycemia-related fetal growth differences [41, 42].

Despite the fact that South Asian women have higher fasting and postload glucose levels and are more likely to develop GDM [11], their infants weigh on average less at birth than infants of White European mothers. Here, we show that the fetal growth difference between South Asians and White Europeans is substantial as, on average, infants of South Asian women with GDM were even smaller across gestation than infants of White Europeans without GDM. There are a number of implications that follow from this. First, this could result in South Asian fetuses and infants with glycemia-related pathological fetal growth not being identified as such. This can be seen by the proportions identified as LGA based on birth weight in this study. Applying the Intergrowth-21st standards, which assess the deviation in birth weight from an ideal size that would be obtained under extreme healthy pregnancy conditions (no complications, within a tightly defined age, height and BMI range, and healthy behaviors such as not smoking in pregnancy), showed higher proportions of LGA in infants of mothers with GDM compared to those without GDM in both ethnicities and also lower rates of LGA in South Asians compared with White Europeans, consistent with the differences in fetal growth that we find by GDM and ethnicity. By contrast, customized reference charts (which assume that maternal ethnicity, as well as height, weight, and parity are physiological causes of variation in birthweight and remove the effects of these characteristics on SGA and LGA) removed the ethnic difference in LGA, as expected given the standardization on ethnicity. Whatever standard or reference charts are used, it may be that lower thresholds for fetal growth and birth weight are necessary to identify South Asians at risk of LGA and potential future risk of obesity. However, such an approach could risk missing cases of SGA and the use of ethnic specific thresholds would only be justified if this would also improve the prediction of adverse neonatal outcomes related to SGA, which remains to be determined. In light of the ongoing debate around universal fetal growth standards vs. reference charts (whether customized or not), further studies addressing this important knowledge gap are warranted [43]. Second, our findings suggest that routine fetal ultrasound measures may be inadequate for the assessment of GDM pathological effects if these are largely driven by other anthropometric measures. Infants born to mothers with GDM have a higher fat to lean body mass [44, 45] and a predisposition to fat over fat-free mass has also been demonstrated in utero from 20 weeks’ gestation [46, 47]. Evidence from the BiB cohort further suggests that higher cord blood leptin levels, a proxy for fetal fat mass, in South Asian compared with White European infants is largely driven by the ethnic difference in maternal glucose levels [23]. Taken together, these data indicate that more detailed fetal body composition measures (distinguishing fetal fat from lean mass trajectories) may be important in identifying pathological effects of gestational hyperglycemia and to ascertain whether “normal” weight South Asian infants of GDM pregnancies might be a group at risk of adverse perinatal and long-term metabolic health.

Strengths of this study are the large sample size, repeat ultrasound measurements of fetal growth from 12/16 weeks of gestation to birth, and the ability to examine joint effects of GDM and ethnicity. Our study was embedded in routine clinical practice with universal OGTT diagnostic testing, which allowed us to determine associations with gestational hyperglycemia across the entire glucose range. Third trimester ultrasound scans, however, were only available for a subset of the cohort. Nonetheless, ~ 30% of all third trimester scans were research scans and not done because of clinical indication, almost all infants had birth measurements as the fetal growth endpoint and exclusion of singletons with ≥ 4 ultrasound scans (which addresses the impact of clinical indications associated with multiple repeat scans) did not change the results. Moreover, all analyses were adjusted for a wide range of maternal characteristics, some of which were also predictive of having an additional third trimester scan. This means that the “missing at random” assumption is plausible, minimizing any potential bias.

Gestational age assessment in our study was based on fetal biometry which relies on the assumption that variation between fetuses is negligible during the dating period. This could have resulted in an underestimation of fetal size variation in early pregnancy and consequent inability to detect associations at this time, especially where fetal HC was used for dating. However, only 22% of all participants had missing CRL data, and it is unlikely that the use of HC for dating has resulted in major bias as mean gestational age at delivery, birth weight, GDM, and fasting and postload glucose were similar between those whose dates were derived from CRL and those from HC. However, the ethnic imbalance in dating method used could have masked the ethnic difference in fetal HC trajectory observed, especially during early gestation when fetal HC measurements are more closely correlated to the dating measure. Finally, we cannot exclude the possibility of a systematic over- or underestimation of early fetal size differences between women with and without GDM. This is an inherent source of potential bias related to the fact that pregnancy dating is performed without knowing whether or not a woman will or will not experience GDM. As fetal size is used to allocate a gestational age, a lower or higher gestational age may have been assigned to GDM cases if their fetus was already smaller or larger than fetuses of women without GDM at the dating scan. The consequence of pregnancy dating being pushed backward or forward in these women is that the actual size of their fetus at a given gestational age in early pregnancy (when fetal anthropometric measures are closely correlated to CRL and HC measures used for dating) could have been overestimated or underestimated relative to the fetal size of normoglycemic women. Finally, we were unable to assess associations with early pregnancy glucose, as this was not measured in the BiB cohort.

## Conclusions

GDM is associated with fetal growth deviation in early pregnancy prior to diagnosis in both ethnic groups. This emphasizes the need for novel strategies to diagnose hyperglycemia-related growth accelerations earlier than current methods. Consistent with the hypothesis that South Asian fetuses adapt to generations of maternal undernutrition by compromising growth and development of abdominal visceral organs with relative sparing of the fetal brain, we found greater ethnic differences between AC and EFW than with HC, but further research is required to understand ethnic differences in fetal growth of fat, lean, and skeletal mass. Furthermore, as fetal growth trajectories differ considerably by ethnicity and this could potentially mask the detection of hyperglycemia-related pathological effects, further work is now needed to understand how this may influence perinatal and offspring health.

## Additional files

Additional file 1: Supplementary methods including model specification of fetal growth trajectory analyses and details on multivariable multiple imputation of missing covariate data. (DOCX 5508 kb)

Additional file 2: Supplementary tables and figures including detailed information on study populations, additional data supporting our findings, and results from sensitivity analyses. (DOCX 25 kb)

## Abbreviations

AC: Abdominal circumference
BiB: Born in Bradford
BMI: Body mass index
BRI: Bradford Royal Infirmary
CRL: Crown rump length
EFW: Estimated fetal weight
FL: Femur length
GDM: Gestational diabetes
HC: Head circumference
IQR: Interquartile range
NHS: National Health Service
NICE: National Institute for Health and Clinical Excellence
OGTT: Oral glucose tolerance test

## References

[1] Lawlor DA, Lichtenstein P, Langstrom N (2011). Association of maternal diabetes mellitus in pregnancy with offspring adiposity into early adulthood: sibling study in a prospective cohort of 280,866 men from 248,293 families. Circulation.

[2] Moser K, Stanfield KM, Leon DA (2008). Birthweight and gestational age by ethnic group, England and Wales 2005: introducing new data on births. Health Stat Q.

[3] West J, Lawlor DA, Fairley L, Bhopal R, Cameron N, McKinney PA (2013). UK-born Pakistani-origin infants are relatively more adipose than white British infants: findings from 8704 mother-offspring pairs in the Born-in-Bradford prospective birth cohort. J Epidemiol Community Health.

[4] Kiserud T, Piaggio G, Carroli G, Widmer M, Carvalho J, Neerup Jensen L (2017). The World Health Organization fetal growth charts: a multinational longitudinal study of ultrasound biometric measurements and estimated fetal weight. PLoS Med.

[5] Buck Louis GM, Grewal J, Albert PS, Sciscione A, Wing DA, Grobman WA (2015). Racial/ethnic standards for fetal growth: the NICHD Fetal Growth Studies. Am J Obstet Gynecol.

[6] Norris T, Tuffnell D, Wright J, Cameron N (2014). Modelling foetal growth in a bi-ethnic sample: results from the Born in Bradford (BiB) birth cohort. Ann Hum Biol.

[7] Farrar D, Simmonds M, Griffin S, Duarte A, Lawlor DA, Sculpher M (2016). The identification and treatment of women with hyperglycaemia in pregnancy: an analysis of individual participant data, systematic reviews, meta-analyses and an economic evaluation. Health Technol Assess.

[8] Sovio U, Murphy HR, Smith GC (2016). Accelerated fetal growth prior to diagnosis of gestational diabetes mellitus: a prospective cohort study of nulliparous women. Diabetes Care.

[9] Crowther CA, Hiller JE, Moss JR, McPhee AJ, Jeffries WS, Robinson JS (2005). Effect of treatment of gestational diabetes mellitus on pregnancy outcomes. N Engl J Med.

[10] Landon MB, Spong CY, Thom E, Carpenter MW, Ramin SM, Casey B (2009). A multicenter, randomized trial of treatment for mild gestational diabetes. N Engl J Med.

[11] Farrar D, Fairley L, Santorelli G, Tuffnell D, Sheldon TA, Wright J (2015). Association between hyperglycaemia and adverse perinatal outcomes in south Asian and white British women: analysis of data from the Born in Bradford cohort. Lancet Diabetes Endocrinol.

[12] Metzger BE, Lowe LP, Dyer AR, Trimble ER, Chaovarindr U, HAPO, Study Cooperative Research Group (2008). Hyperglycemia and adverse pregnancy outcomes. N Engl J Med.

[13] Farrar D, Simmonds M, Bryant M, Sheldon TA, Tuffnell D, Golder S (2016). Hyperglycaemia and risk of adverse perinatal outcomes: systematic review and meta-analysis. BMJ.

[14] Wright J, Small N, Raynor P, Tuffnell D, Bhopal R, Cameron N (2013). Cohort profile: the Born in Bradford multi-ethnic family cohort study. Int J Epidemiol.

[15] Bradford District Infant Mortality Commission: Summary Report. 2006. https://www.bradford.gov.uk/media/1881/infant_mortality_report.pdf. Accessed 30 Apr 2018.

[16] National Institute for Health and Clinical Excellence. Antenatal Care: NICE clinical guideline 62. https://www.nice.org.uk/guidance/cg62. Accessed 30 Apr 2018.

[17] Loughna Pam, Chitty Lyn, Evans Tony, Chudleigh Trish (2009). Fetal Size and Dating: Charts Recommended for Clinical Obstetric Practice. Ultrasound.

[18] Hadlock FP, Harrist RB, Sharman RS, Deter RL, Park SK (1985). Estimation of fetal weight with the use of head, body, and femur measurements—a prospective study. Am J Obstet Gynecol.

[19] Mirghani HM, Weerasinghe S, Ezimokhai M, Smith JR (2005). Ultrasonic estimation of fetal weight at term: an evaluation of eight formulae. J Obstet Gynaecol Res.

[20] West J, Lawlor DA, Fairley L, Wright J (2014). Differences in socioeconomic position, lifestyle and health-related pregnancy characteristics between Pakistani and White British women in the Born in Bradford prospective cohort study: the influence of the woman’s, her partner’s and their parents’ place of birth. BMJ Open.

[21] Villar J, Cheikh Ismail L, Victora CG, Ohuma EO, Bertino E, Altman DG (2014). International standards for newborn weight, length, and head circumference by gestational age and sex: the Newborn Cross-Sectional Study of the INTERGROWTH-21st Project. Lancet.

[22] Gardosi J, Chang A, Kalyan B, Sahota D, Symonds EM (1992). Customised antenatal growth charts. Lancet.

[23] Lawlor DA, West J, Fairley L, Nelson SM, Bhopal RS, Tuffnell D (2014). Pregnancy glycaemia and cord-blood levels of insulin and leptin in Pakistani and white British mother-offspring pairs: findings from a prospective pregnancy cohort. Diabetologia.

[24] Leckie G, Charlton C. Runmlwin: a program to run the MLwiN Multilevel Modeling Software from within Stata. J Stat Softw. 2013;52:1–40.

[25] Sletner L, Rasmussen S, Jenum AK, Nakstad B, Jensen OH, Vangen S (2015). Ethnic differences in fetal size and growth in a multi-ethnic population. Early Hum Dev.

[26] Krishnaveni GV, Yajnik CS (2017). Developmental origins of diabetes-an Indian perspective. Eur J Clin Nutr.

[27] Yajnik CS, Fall CH, Coyaji KJ, Hirve SS, Rao S, Barker DJ (2003). Neonatal anthropometry: the thin-fat Indian baby. The Pune Maternal Nutrition Study. Int J Obes Relat Metab Disord.

[28] Krishnaveni GV, Hill JC, Veena SR, Leary SD, Saperia J, Chachyamma KJ (2005). Truncal adiposity is present at birth and in early childhood in South Indian children. Indian Pediatr.

[29] Stanfield KM, Wells JC, Fewtrell MS, Frost C, Leon DA (2012). Differences in body composition between infants of South Asian and European ancestry: the London Mother and Baby Study. Int J Epidemiol.

[30] D'Angelo S, Yajnik CS, Kumaran K, Joglekar C, Lubree H, Crozier SR (2015). Body size and body composition: a comparison of children in India and the UK through infancy and early childhood. J Epidemiol Community Health.

[31] Catalano PM, McIntyre HD, Cruickshank JK, McCance DR, Dyer AR, Metzger BE (2012). The hyperglycemia and adverse pregnancy outcome study: associations of GDM and obesity with pregnancy outcomes. Diabetes Care.

[32] Sletner L, Jenum AK, Yajnik CS, Morkrid K, Nakstad B, Rognerud-Jensen OH (2017). Fetal growth trajectories in pregnancies of European and South Asian mothers with and without gestational diabetes, a population-based cohort study. PLoS One.

[33] Hammoud NM, Visser GH, Peters SA, Graatsma EM, Pistorius L, de Valk HW (2013). Fetal growth profiles of macrosomic and non-macrosomic infants of women with pregestational or gestational diabetes. Ultrasound Obstet Gynecol.

[34] Mulder EJ, Visser GH (1991). Growth and motor development in fetuses of women with type-1 diabetes. I. Early growth patterns. Early Hum Dev.

[35] Pedersen JF, Molsted-Pedersen L (1979). Early growth retardation in diabetic pregnancy. Br Med J.

[36] Desoye G (2018). The human placenta in diabetes and obesity: friend or foe? The 2017 Norbert Freinkel award lecture. Diabetes Care.

[37] Sattar N, Greer IA (2002). Pregnancy complications and maternal cardiovascular risk: opportunities for intervention and screening?. BMJ.

[38] Hedderson MM, Darbinian JA, Quesenberry CP, Ferrara A (2011). Pregravid cardiometabolic risk profile and risk for gestational diabetes mellitus. Am J Obstet Gynecol.

[39] Catalano PM, Tyzbir ED, Wolfe RR, Calles J, Roman NM, Amini SB (1993). Carbohydrate metabolism during pregnancy in control subjects and women with gestational diabetes. Am J Phys.

[40] Catalano PM, Huston L, Amini SB, Kalhan SC (1999). Longitudinal changes in glucose metabolism during pregnancy in obese women with normal glucose tolerance and gestational diabetes mellitus. Am J Obstet Gynecol.

[41] Fleming TP, Watkins AJ, Velazquez MA, Mathers JC, Prentice AM, Stephenson J (2018). Origins of lifetime health around the time of conception: causes and consequences. Lancet.

[42] Catalano PM (2014). Trying to understand gestational diabetes. Diabet Med.

[43] Iliodromiti S, Mackay DF, Smith GC, Pell JP, Sattar N, Lawlor DA (2017). Customised and noncustomised birth weight centiles and prediction of stillbirth and infant mortality and morbidity: a cohort study of 979,912 term singleton pregnancies in Scotland. PLoS Med.

[44] Catalano PM, Thomas A, Huston-Presley L, Amini SB (2003). Increased fetal adiposity: a very sensitive marker of abnormal in utero development. Am J Obstet Gynecol.

[45] Nasrat H, Abalkhail B, Fageeh W, Shabat A, el Zahrany F (1997). Anthropometric measurement of newborns of gestational diabetic mothers: does it indicate disproportionate fetal growth?. J Matern Fetal Med.

[46] Larciprete G, Valensise H, Vasapollo B, Novelli GP, Parretti E, Altomare F (2003). Fetal subcutaneous tissue thickness (SCTT) in healthy and gestational diabetic pregnancies. Ultrasound Obstet Gynecol.

[47] Venkataraman H, Ram U, Craik S, Arungunasekaran A, Seshadri S, Saravanan P (2017). Increased fetal adiposity prior to diagnosis of gestational diabetes in South Asians: more evidence for the ‘thin-fat’ baby. Diabetologia.

