# Supplementary material for: Gestational diabetes and ultrasound-assessed fetal growth in South Asian and White European women: findings from a prospective pregnancy cohort
Source: BMC Med. 2018 Nov 6;16:203. doi: 10.1186/s12916-018-1191-7 (PMC6219043; doi:10.1186/s12916-018-1191-7)
Supplement: Supplementary file 1 — Supplementary methods including model specification of fetal growth trajectory analyses and details on multivariable multiple imputation of missing covariate data. (DOCX 5508 kb) [file 12916_2018_1191_MOESM1_ESM.docx]

**Supplementary Tables**

**Table S1.** Descriptive statistics of the repeat ultrasound and birth anthropometric measurements in the analysis cohort.

**Table S2.** Comparison of observed means of fetal size with those predicted by the multilevel fractional polynomial models.

**Table S3.** Maternal and infant characteristics of the analysis cohort and larger eligible cohort of singletons.

**Table S4.** Maternal and infant characteristics in the analysis cohort by availability of 3^rd^ trimester ultrasound scan data.

**Table S5.** Maternal and infant characteristics in the anaylsis cohort by ultrasound measurement used for dating.

**Table S6.** Predicted differences in mean fetal size across gestation by fasting glucose and 2-hour postload glucose levels.

**Table S7.** Predicted differences in mean fetal size across gestation by ethnicity and gestational diabetes – model 1.

**Table S8.** Predicted differences in mean fetal size across gestation by ethnicity and gestational diabetes– complete case analyses.

**Table S9.** Predicted differences in mean fetal size across gestation by ethnicity and gestational diabetes - analyses restricted to singletons of women with ≤ 4 repeat ultrasound scans.

**Table S10.** Predicted differences in mean fetal size across gestation by ethnicity and gestational diabetes – analyses restricted to preganncies with an OGTT between 24 and 30 weeks.

**Supplementary Figures**

**Figure S1.** Flow chart of the study population.

**Figure S2.** Best-fitting growth trajectories for each fetal parameter identified by multilevel fractional polynomial models.

**Figure S3.** Average predicted fetal growth trajectories, stratified by ethnicity.

**Figure S4.** Average predicted fetal growth trajectories, stratified by gestational diabetes.

**Figure S5.** Associations of gestational fasting glucose with fetal size across gestation in White Europeans and South Asians.

**Figure S6.** Associations of gestational 2-hour postload glucose with fetal size across gestation in White Europeans and South Asians.

**Figure S7.** Average predicted fetal growth trajectories, stratified by ethnicity and gestational diabetes.

**Table S1.** Descriptive statistics of the repeat ultrasound and birth anthropometric measurements in the analysis cohort.

| **Measure** | **N with at least one measure** | **Median (range) no. of**  **measurements per individual** | **Total no. of**  **measurements** | **Mean (SD)** | **Mean (SD) gestational age**  **at measurement (wks)** | **No (%) of birth measurements** |
| --- | --- | --- | --- | --- | --- | --- |
| Head circumference (mm) | 10700 | 3 (1;12) | 33118 | 260 (83) | 29.1 (9.1) | 9871 (29.8) |
| Femur length (mm) | 10689 | 3 (1;11) | 23694 | 44 (17) | 25.1 (7.3) | 0 (0) |
| Abdominal circumference (mm) | 10520 | 3 (1;10) | 28096 | 254 (73) | 31.1 (8.1) | 9371 (33.4) |
| Estimated fetal weight (g) | 10701 | 3 (1;10) | 28464 | 2020 (1267) | 31.5 (8.1) | 10688 (37.5) |

Abbreviations: SD = standard deviation; wks = weeks.

**Table S2.** Comparison of observed means of fetal size with those predicted by the multilevel fractional polynomial model.

|  |  |  | **Observed** | | **Predicted** | | **Difference** | | **95% limits of agreement** | |
| --- | --- | --- | --- | --- | --- | --- | --- | --- | --- | --- |
| **Measure** | **Gestational age** | **No. of obs** | **Mean** | **SD** | **Mean** | **SD** | **Mean** | **SD** | **P5** | **P95** |
| Head circumference (mm) | 12-15 wks | 1604 | 96.7 | 15.3 | 96.9 | 14.7 | -0.2 | 2.4 | -3.5 | 3.4 |
|  | 16-19 wks | 4155 | 165.3 | 14.6 | 164.6 | 13.9 | 0.7 | 2.9 | -3.7 | 5.5 |
|  | 20-23 wks | 7763 | 181.8 | 11.9 | 182.3 | 11.0 | -0.5 | 3.0 | -5.3 | 4.3 |
|  | 24-27 wks | 623 | 241.2 | 18.6 | 241.0 | 16.6 | 0.2 | 4.8 | -7.9 | 7.7 |
|  | 28-31 wks | 2570 | 284.0 | 13.2 | 282.6 | 11.3 | 1.4 | 5.2 | -6.6 | 9.9 |
|  | 32-35 wks | 4772 | 313.5 | 11.9 | 313.7 | 10.2 | -0.2 | 5.1 | -8.8 | 8.1 |
|  | 36-39 wks | 6807 | 336.4 | 14.1 | 337.0 | 10.5 | -0.6 | 5.7 | -9.3 | 8.6 |
|  | >39 wks | 4824 | 347.5 | 13.2 | 347.0 | 9.1 | 0.5 | 5.3 | -7.7 | 8.7 |
| Femur length (mm) | 12-15 wks | 1350 | 13.7 | 3.6 | 13.8 | 3.5 | -0.1 | 1.1 | -1.7 | 1.8 |
|  | 16-19 wks | 4255 | 29.7 | 3.7 | 29.5 | 3.3 | 0.2 | 1.1 | -1.5 | 2.1 |
|  | 20-23 wks | 7884 | 33.7 | 2.9 | 33.8 | 2.5 | -0.1 | 1.0 | -1.7 | 1.6 |
|  | 24-27 wks | 629 | 47.1 | 4.3 | 47.2 | 3.7 | -0.1 | 1.3 | -2.1 | 1.8 |
|  | 28-31 wks | 2589 | 57.0 | 3.1 | 56.9 | 2.6 | 0.1 | 1.3 | -2.0 | 2.2 |
|  | 32-35 wks | 4731 | 64.6 | 2.9 | 64.5 | 2.3 | 0.1 | 1.2 | -1.8 | 1.9 |
|  | 36-39 wks | 2220 | 69.5 | 2.9 | 69.6 | 2.4 | -0.1 | 1.1 | -1.9 | 1.8 |
|  | >39 wks | 36 | 72.0 | 2.8 | 72.4 | 2.3 | -0.4 | 0.9 | -2.1 | 1.1 |
| Abdominal circumference (mm) | 16-19 wks | 2686 | 145.6 | 8.7 | 143.8 | 5.1 | 1.8 | 4.5 | -5.1 | 9.0 |
|  | 20-23 wks | 5847 | 155.1 | 11.8 | 156.0 | 9.3 | -0.9 | 4.5 | -7.8 | 6.7 |
|  | 24-27 wks | 554 | 213.0 | 19.1 | 212.8 | 17.2 | 0.2 | 5.7 | -9.1 | 10.0 |
|  | 28-31 wks | 2625 | 255.9 | 17.6 | 256.7 | 15.1 | -0.8 | 6.2 | -10.6 | 9.4 |
|  | 32-35 wks | 4942 | 292.6 | 19.1 | 291.4 | 14.2 | 1.2 | 8.5 | -12.1 | 14.6 |
|  | 36-39 wks | 6845 | 311.5 | 23.9 | 312.4 | 13.1 | -0.9 | 15.4 | -27.3 | 22.0 |
|  | >39 wks | 4597 | 318.3 | 24.2 | 318.2 | 9.9 | 0.1 | 16.1 | -26.6 | 25.8 |
| Estimated fetal weight (g) | 16-19 wks | 2611 | 312 | 36 | 300 | 26 | 12 | 14 | -8 | 36 |
|  | 20-23 wks | 5621 | 368 | 72 | 375 | 67 | -7 | 18 | -35 | 21 |
|  | 24-27 wks | 552 | 888 | 209 | 901 | 195 | -13 | 47 | -85 | 62 |
|  | 28-31 wks | 2561 | 1515 | 255 | 1514 | 229 | 1 | 78 | -120 | 132 |
|  | 32-35 wks | 4765 | 2248 | 338 | 2213 | 288 | 35 | 118 | -152 | 232 |
|  | 36-39 wks | 7153 | 3028 | 474 | 3026 | 390 | 2 | 168 | -270 | 275 |
|  | >39 wks | 5201 | 3435 | 455 | 3474 | 336 | -39 | 168 | -315 | 233 |

Comparison of predicted values of fetal size by the multilevel fractional polynomial models with the actual values observed; The 95% limits of agreement refer to the range within which 95% of the differences between the predicted and observed values lie. Abbreviations: SD = standard deviation; No. of obs = number of observed measurements; p5 = 5^th^ percentile and p95 = 95^th^ percentile.

**Table S3.** Maternal and infant characteristics of the analysis cohort and larger eligible cohort of singletons.

|  | **Analysis cohort**  **(N = 10705)** | **Eligible cohort**  **(N = 11697)** |
| --- | --- | --- |
| **Infant characteristics** |  |  |
| Sex, % (N) |  |  |
| Male | 51.6 (5524) | 51.5 (6018) |
| Female | 48.4 (5181) | 48.6 (5678) |
| Missing |  | 0.01 (1) |
| Ethnicity by country, % (N) |  |  |
| White British | 41.1 (4396) | 41.2 (4813) |
| White other | 3.3 (351) | 3.4 (397) |
| Pakistani | 48.4 (5181) | 48.1 (5634) |
| Indian | 4.0 (432) | 4.1 (478) |
| Bangladeshi | 3.2 (345) | 3.2 (375) |
| Birth outcome, % (N) |  |  |
| Livebirth | 99.5 (10656) | 99.4 (11630) |
| Stillbirth | 0.5 (49) | 0.6 (67) |
| 5-min Apgar score (≤ 5), % (N) |  |  |
| No | 99.1 (10447) | 99.0 (11162) |
| Yes | 0.9 (90) | 1.0 (108) |
| Missing | 1.6 (168) | 3.7 (427) |
| Hypothermia (< 36.5°C), % (N) |  |  |
| No | 92.9 (8636) | 92.8 (9211) |
| Yes | 7.1 (663) | 7.2 (712) |
| Missing | 13.1 (1406) | 15.2 (1774) |
| Birthweight (gram), mean (SD) | 3234 (541) | 3233 (546) |
| Missing, % (N) | 0.1 (10) | 2.0 (229) |
| Gestational age at birth (weeks), mean (SD) | 39.2 (1.7) | 39.2 (1.8) |
| Missing, % (N) | 0.1 (10) | 2.0 (228) |
| Intergrowth birth weight centiles  (full cohort analyses), % (N) * |  |  |
| SGA (< 10th) | 12.5 (1339) | 12.4 (1418) |
| LGA (> 90th) | 10.5 (1126) | 10.7 (1225) |
| Missing | 0.1 (10) | 2.0 (239) |
| Intergrowth birth weight centiles  (restricted analyses), % (N) * |  |  |
| SGA (< 10th) | 12.1 (1005) | 11.9 (1052) |
| LGA (> 90th) | 10.6 (881) | 10.8 (948) |
| Missing | 22.3 (2392) | 24.6 (2881) |
| Customized birth weight centiles, % (N) ** |  |  |
| SGA (< 10th) | 15.7 (1304) | 15.6 (1374) |
| LGA (> 90th) | 5.5 (455) | 5.6 (489) |
| Missing | 22.3 (2392) | 24.6 (2881) |
| **Maternal characteristics** |  |  |
| Maternal age at delivery (years), mean (SD) | 27.4 (5.5) | 27.4 (5.5) |
| Multiparity, % (N) |  |  |
| Primiparous | 39.4 (4081) | 39.4 (4352) |
| 1 | 28.9 (2990) | 28.8 (3179) |
| 2 | 17.2 (1783) | 17.2 (1907) |
| ≥ 3 | 14.5 (1503) | 14.7 (1621) |
| Missing | 3.3 (348) | 5.5 (638) |

**Table S3.** *Continued*

|  | **Analysis cohort**  **(N = 10705)** | **Eligible cohort**  **(N = 11697)** |
| --- | --- | --- |
| **Maternal characteristics** |  |  |
| Height (cm), mean (SD) | 161.6 (6,5) | 161.6 (6.5) |
| Missing, % (N) | 15.8 (1688) | 16.3 (1908) |
| Body mass index (kg/m2), mean (SD) | 26.0 (5.7) | 26.0 (5.7) |
| Missing, % (N) | 17.3 (1855) | 20.2 (2362) |
| Education, % (N) |  |  |
| < 5 GCSEs | 22.3 (2007) | 22.3 (2183) |
| 5 GCSEs | 32.4 (2917) | 32.0 (3132) |
| A-level | 14.9 (1339) | 15.0 (1465) |
| Higher than A-level | 24.9 (2246) | 25.3 (2474) |
| Other | 5.6 (506) | 5.5 (541) |
| Missing | 15.8 (1690) | 16.3 (1902) |
| Smoking during pregnancy, % (N) |  |  |
| No | 83.3 (7653) | 83.2 (8306) |
| Yes | 16.7 (1530) | 16.8 (1672) |
| Missing | 14.2 (1522) | 14.7 (1719) |
| Alcohol use during pregnancy, % (N) |  |  |
| No | 68.7 (6307) | 68.7 (6849) |
| Yes | 31.3 (2869) | 31.3 (3121) |
| Missing | 14.3 (1529) | 14.8 (1727) |
| Gestational diabetes, % (N) |  |  |
| No | 92.2 (9873) | 92.4 (10113) |
| Yes | 7.8 (832) | 7.6 837) |
| Missing | 0.0 (0) | 6.4 (747) |
| Fasting glucose (mmol/L), mean (SD) *** | 4.47 (0.40) | 4.47 (0.40) |
| Missing, % (N) | 8.6 (916) | 9.5 (958) |
| 2-hour postload glucose (mmol/L), mean (SD) *** | 5.39 (1.05) | 5.39 (1.05) |
| Missing, % (N) | 8.7 (929) | 9.6 (973) |
| Hypertensive disorders of pregnancy, % (N) |  |  |
| No | 94.1 (10071) | 94.1 (10324) |
| Yes | 5.9 (634) | 5.9 (649) |
| Missing | 0.0 (0) | 6.2 (724) |

Abbreviations: SD = standard deviation; GCSE = General Certificate of Secondary Education. The eligible cohort includes White European and South Asian singletons without known congenital abnormalities, irrespective of available data on gestational diabetes and ultrasound scans. * SGA and LGA defined based on noncustomized birth weight centiles (standardized on sex and gestational age only) according to Intergrowth-21^st^ standards. ** SGA and LGA defined based on GROW customized birth weight centiles (standardized on sex, gestational age, maternal parity, height and weight). For the Intergrowth-21st (noncustomised) charts we present results for the maximal sample and for the smaller sample (with no missing data on maternal parity, height and weight) used for comparison with customized birth weight centiles.

*** In women without a GDM diagnosis.

**Table S4.** Maternal and infant characteristics by availability of 3^rd^ trimester ultrasound scan data.

|  | **3^rd^ trimester ultrasound scan data** | |
| --- | --- | --- |
|  | **No**  **(N = 5446)** | **Yes**  **(N = 5259)** |
| **Infant characteristics** |  |  |
| Sex, % (N) |  |  |
| Male | 52.5 (2858) | 50.7 (2666) |
| Female | 47.5 (2588) | 49.3 (2593) |
| Ethnicity, % (N) |  |  |
| White European | 44.7 (2434) | 44.0 (2313) |
| South Asian | 55.3 (3012) | 56.0 (2946) |
| Birthweight (gram), mean (SD) | 3299 (516) | 3168 (558) |
| Gestational age at birth (weeks), mean (SD) | 39.4 (1.8) | 39.0 (1.7) |
| **Maternal characteristics** |  |  |
| Age at delivery (years), mean (SD) | 26.9 (5.4) | 28.0 (5.7) |
| Multiparity, % (N) |  |  |
| Primiparous | 43.0 (2258) | 35.7 (1823) |
| 1 | 28.3 (1488) | 29.5 (1502) |
| 2 | 16.1 (844) | 18.4 (939) |
| ≥ 3 | 12.7 (667) | 16.4 (836) |
| Height (cm), mean (SD) | 162.0 (6.4) | 161.1 (6.5) |
| Body mass index (kg/m^2^), mean (SD) | 25.6 (5) | 26.4 (6) |
| Education, % (N) |  |  |
| < 5 GCSEs | 21.7 (978) | 22.8 (1029) |
| 5 GCSEs | 32.7 (1473) | 32.0 (1444) |
| A-level | 14.6 (657) | 15.1 (682) |
| Higher than A-level | 25.1 (1131) | 24.7 (1115) |
| Other | 5.9 (266) | 5.3 (240) |
| Smoking during pregnancy, % (N) |  |  |
| No | 84.0 (3852) | 82.7 (3801) |
| Yes | 16.0 (735) | 17.3 (795) |
| Alcohol use during pregnancy, % (N) |  |  |
| No | 67.5 (3096) | 69.9 (3211) |
| Yes | 32.5 (1488) | 30.1 (1381) |
| Gestational diabetes, % (N) |  |  |
| No | 99.6 (5423) | 84.6 (4450) |
| Yes | 0.4 (23) | 15.4 (809) |
| Fasting glucose (mmol/L), mean (SD) * | 4.5 (0.4) | 4.5 (0.4) |
| 2-hour postload glucose (mmol/), mean (SD) * | 5.4 (1.1) | 5.4 (1.0) |
| Hypertensive disorder of pregnancy, % (N) |  |  |
| No | 95.8 (5217) | 92.3 (4854) |
| Yes | 4.2 (229) | 7.7 (405) |

Abbreviations: SD = standard deviation; GCSE = General Certificate of Secondary Education. * In women without a GDM diagnosis.

**Table S5.** Maternal and infant characteristics by ultrasound measurement used for dating.

|  | **CRL for dating**  **(N = 8364)** | **Fetal HC for dating**  **(N = 2341)** |
| --- | --- | --- |
| **Infant characteristics** |  |  |
| Sex, % (N) |  |  |
| Male | 51.0 (4268) | 53.7 (1256) |
| Female | 49.0 (4096) | 46.3 (1085) |
| Ethnicity, % (N) |  |  |
| White European | 46.8 (3917) | 35.5 (830) |
| South Asian | 53.2 (4447) | 64.5 (1511) |
| Birthweight (gram), mean (SD) | 3240 (539) | 3214 (546) |
| Gestational age at birth (weeks), mean (SD) | 39.2 (1.7) | 39.2 (1.7) |
| **Maternal characteristics** |  |  |
| Age at delivery (years), mean (SD) | 27.5 (5.5) | 27.2 (5.8) |
| Multiparity, % (N) |  |  |
| Primiparous | 39.6 (3216) | 38.6 (865) |
| 1 | 29.7 (2411) | 25.8 (579) |
| 2 | 17.0 (1381) | 17.9 (402) |
| ≥ 3 | 13.7 (1109) | 17.6 (394) |
| Height (cm), mean (SD) | 161.7 (6.5) | 161.1 (6.5) |
| Body mass index (kg/m^2^), mean (SD) | 26.0 (5.7) | 26.1 (5.8) |
| Education, % (N) |  |  |
| < 5 GCSEs | 20.7 (1479) | 28.2 (528) |
| 5 GCSEs | 32.4 (2314) | 32.2 (603) |
| A-level | 15.4 (1102) | 12.7 (237) |
| Higher than A-level | 25.7 (1834) | 22.0 (412) |
| Other | 5.8 (414) | 4.9 (92) |
| Smoking during pregnancy, % (N) |  |  |
| No | 82.9 (6033) | 84.9 (1620) |
| Yes | 17.1 (1242) | 15.1 (288) |
| Alcohol use during pregnancy, % (N) |  |  |
| No | 67.4 (4899) | 73.8 (1408) |
| Yes | 32.6 (2370) | 26.2 (499) |
| Gestational diabetes, % (N) |  |  |
| No | 92.2 (7709) | 92.4 (2164) |
| Yes | 7.8 (655) | 7.6 (177) |
| Fasting glucose (mmol/L), mean (SD) * | 4.5 (0.4) | 4.5 (0.4) |
| 2-hour postload glucose (mmol/), mean (SD) * | 5.4 (1.1) | 5.4 (1.0) |
| Hypertensive disorder of pregnancy, % (N) |  |  |
| No | 93.9 (7854) | 94.7 (2217) |
| Yes | 6.1 (510) | 5.3 (124) |

Abbreviations: SD = standard deviation; GCSE = General Certificate of Secondary Education. * In women without a GDM diagnosis.

**Table S6.** Predicted differences in mean fetal size across gestation by fasting glucose and 2-hour postload glucose levels.

|  |  | **Predicted mean difference (95% CI)** | | | | | | | |
| --- | --- | --- | --- | --- | --- | --- | --- | --- | --- |
| **HC (mm)** | **N** | **12 wks** | **16 wks** | **20 wks** | **24 wks** | **28 wks** | **32 wks** | **36 wks** | **40 wks** |
| Fasting glucose |  |  |  |  |  |  |  |  |  |
| Q1 (≤ 4.1 mmol/L) | 1885 | REF | REF | REF | REF | REF | REF | REF | REF |
| Q2 (4.2-4.3 mmol/L) | 1907 | 0.2 (-0.6; 1.0) | 0.2 (-0.3; 0.7) | 0.2 (-0.3; 0.6) | 0.2 (-0.3; 0.7) | 0.3 (-0.4; 0.9) | 0.4 (-0.3; 1.0) | 0.5 (-0.2; 1.2) | 0.7 (-0.1; 1.5) |
| Q3 (4.4.-4.5 mmol/L) | 1877 | -0.2 (-1.0; 0.6) | -0.0 (-0.6; 0.5) | 0.1 (-0.3; 0.6) | 0.3 (-0.2; 0.8) | 0.5 (-0.2; 1.1) | 0.6 (-0.1; 1.3) | 0.8 (0.1; 1.5) | 1.0 (0.2; 1.8) |
| Q4 (4.6-4.8 mmol/L) | 1881 | -0.3 (-1.1; 0.5) | -0.1 (-0.6; 0.5) | 0.2 (-0.3; 0.6) | 0.4 (-0.1; 1.0) | 0.7 (0.0; 1.3) | 1.0 (0.3; 1.7) | 1.3 (0.6; 2.0) | 1.7 (0.9; 2.5) |
| Q5 (4.9-6.0 mmol/L) | 1402 | -0.1 (-1.0; 0.8) | 0.1 (-0.5; 0.7) | 0.3 (-0.2; 0.8) | 0.5 (-0.1; 1.1) | 0.8 (0.0; 1.5) | 1.1 (0.3; 1.9) | 1.5 (0.8; 2.3) | 2.1 (1.2; 3.0) |
| per sd increase | 8952 | -0.1 (-0.4; 0.2) | -0.0 (-0.2; 0.2) | 0.1 (-0.1; 0.2) | 0.2 (0.0; 0.4) | 0.3 (0.1; 0.5) | 0.5 (0.2; 0.7) | 0.6 (0.4; 0.8) | 0.8 (0.5; 1.1) |
| 2-h postload glucose |  |  |  |  |  |  |  |  |  |
| Q1 (≤ 4.5 mmol/L) | 1955 | REF | REF | REF | REF | REF | REF | REF | REF |
| Q2 (4.6-5.1 mmol/L) | 1788 | 0.3 (-0.5; 1.1) | 0.1 (-0.4; 0.6) | -0.1 (-0.5; 0.4) | -0.2 (-0.7; 0.3) | -0.3 (-1.0; 0.3) | -0.4 (-1.1; 0.2) | -0.5 (-1.2; 0.2) | -0.5 (-1.3; 0.3) |
| Q3 (5.2-5.6 mmol/L) | 1637 | 0.3 (-0.6; 1.1) | 0.2 (-0.4; 0.7) | 0.1 (-0.4; 0.5) | 0.0 (-0.6; 0.5) | -0.1 (-0.7; 0.6) | -0.1 (-0.8; 0.6) | 0.0 (-0.7; 0.7) | 0.1 (-0.7; 1.0) |
| Q4 (5.7-6.3 mmol/L) | 1821 | 0.7 (-0.1; 1.5) | 0.5 (0.0; 1.0) | 0.3 (-0.1; 0.8) | 0.2 (-0.3; 0.8) | 0.3 (-0.4; 0.9) | 0.4 (-0.3; 1.1) | 0.7 (0.0; 1.4) | 1.3 (0.4; 2.1) |
| Q5 (6.4-7.7 mmol/L) | 1738 | 0.4 (-0.4; 1.3) | 0.2 (-0.3; 0.8) | 0.1 (-0.4; 0.5) | 0.0 (-0.6; 0.5) | 0.0 (-0.6; 0.7) | 0.3 (-0.4; 1.0) | 0.7 (0.0; 1.4) | 1.4 (0.6; 2.2) |
| per sd increase | 8939 | 0.1 (-0.2; 0.3) | 0.1 (-0.1; 0.2) | 0.0 (-0.1; 0.2) | 0.1 (-0.1; 0.2) | 0.1 (-0.1; 0.3) | 0.2 (0.0; 0.5) | 0.4 (0.2; 0.7) | 0.7 (0.4; 1.0) |
| **FL (mm)** | **N** | **12 wks** | **16 wks** | **20 wks** | **24 wks** | **28 wks** | **32 wks** | **36 wks** | **40 wks** |
| Fasting glucose |  |  |  |  |  |  |  |  |  |
| Q1 (≤ 4.1 mmol/L) | 1886 | REF | REF | REF | REF | REF | REF | REF | REF |
| Q2 (4.2-4.3 mmol/L) | 1901 | 0.2 (-0.1; 0.6) | 0.1 (-0.1; 0.3) | -0.0 (-0.1; 0.1) | -0.0 (-0.2; 0.1) | -0.0 (-0.2; 0.1) | 0.0 (-0.1; 0.2) | 0.2 (0.0; 0.4) | 0.4 (0.0; 0.7) |
| Q3 (4.4.-4.5 mmol/L) | 1875 | -0.1 (-0.4; 0.3) | 0.0 (-0.1; 0.2) | 0.1 (0.0; 0.2) | 0.2 (0.0; 0.3) | 0.2 (0.1; 0.4) | 0.2 (0.0; 0.4) | 0.2 (0.0; 0.4) | 0.2 (-0.2; 0.5) |
| Q4 (4.6-4.8 mmol/L) | 1881 | 0.1 (-0.3; 0.4) | 0.1 (-0.1; 0.2) | 0.1 (-0.1; 0.2) | 0.1 (-0.1; 0.2) | 0.1 (-0.1; 0.2) | 0.1 (-0.1; 0.3) | 0.1 (-0.1; 0.4) | 0.2 (-0.2; 0.5) |
| Q5 (4.9-6.0 mmol/L) | 1400 | 0.1 (-0.3; 0.5) | 0.1 (-0.1; 0.3) | 0.1 (0.0; 0.2) | 0.1 (0.0; 0.3) | 0.2 (0.0; 0.4) | 0.3 (0.1; 0.5) | 0.4 (0.1; 0.6) | 0.5 (0.1; 0.9) |
| per sd increase | 8943 | -0.1 (-0.2; 0.1) | -0.0 (-0.1; 0.1) | 0.0 (0.0; 0.1) | 0.1 (0.0; 0.1) | 0.1 (0.0; 0.2) | 0.1 (0.1; 0.2) | 0.1 (0.0; 0.2) | 0.1 (0.0; 0.3) |
| 2-h postload glucose |  |  |  |  |  |  |  |  |  |
| Q1 (≤ 4.5 mmol/L) | 1953 | REF | REF | REF | REF | REF | REF | REF | REF |
| Q2 (4.6-5.1 mmol/L) | 1788 | 0.1 (-0.3; 0.4) | 0.1 (-0.1; 0.2) | 0.1 (-0.1; 0.2) | 0.1 (-0.1; 0.2) | 0.1 (-0.1; 0.2) | 0.1 (-0.1; 0.3) | 0.1 (-0.1; 0.4) | 0.2 (-0.2; 0.5) |
| Q3 (5.2-5.6 mmol/L) | 1634 | 0.3 (0.0; 0.7) | 0.2 (0.0; 0.4) | 0.1 (0.0; 0.2) | 0.1 (-0.1; 0.2) | 0.1 (-0.1; 0.2) | 0.1 (-0.1; 0.3) | 0.2 (0.0; 0.5) | 0.4 (0.0; 0.8) |
| Q4 (5.7-6.3 mmol/L) | 1820 | 0.0 (-0.3; 0.4) | 0.1 (-0.1; 0.2) | 0.1 (0.0; 0.2) | 0.2 (0.0; 0.3) | 0.2 (0.1; 0.4) | 0.3 (0.1; 0.5) | 0.3 (0.1; 0.6) | 0.4 (0.0; 0.8) |
| Q5 (6.4-7.7 mmol/L) | 1735 | 0.4 (0.0; 0.7) | 0.2 (0.0; 0.4) | 0.1 (0.0; 0.2) | 0.1 (-0.1; 0.2) | 0.2 (0.0; 0.3) | 0.3 (0.2; 0.5) | 0.6 (0.4; 0.9) | 1.0 (0.6; 1.4) |
| per sd increase | 8930 | 0.1 (0.0; 0.2) | 0.1 (0.0; 0.1) | 0.0 (0.0; 0.1) | 0.0 (0.0; 0.1) | 0.1 (0.0; 0.1) | 0.1 (0.1; 0.2) | 0.2 (0.1; 0.3) | 0.4 (0.2; 0.5) |

**Table S6.** *Continued*

|  |  | **Predicted mean difference (95% CI)** | | | | | | | |
| --- | --- | --- | --- | --- | --- | --- | --- | --- | --- |
| **AC (mm)** | **N** | **12 wks** | **16 wks** | **20 wks** | **24 wks** | **28 wks** | **32 wks** | **36 wks** | **40 wks** |
| Fasting glucose |  |  |  |  |  |  |  |  |  |
| Q1 (≤ 4.1 mmol/L) | 1846 | - | REF | REF | REF | REF | REF | REF | REF |
| Q2 (4.2-4.3 mmol/L) | 1876 | - | -0.5 (-1.4; 0.3) | -0.2 (-0.7; 0.4) | 0.3 (-0.5; 1.1) | 0.7 (-0.5; 1.9) | 1.0 (-0.3; 2.3) | 1.1 (-0.1; 2.3) | 0.9 (-0.6; 2.4) |
| Q3 (4.4.-4.5 mmol/L) | 1852 | - | -0.3 (-1.1; 0.6) | 0.0 (-0.5; 0.6) | 0.5 (-0.3; 1.3) | 0.9 (-0.3; 2.1) | 1.3 (-0.1; 2.6) | 1.5 (0.3; 2.7) | 1.5 (0.0; 3.1) |
| Q4 (4.6-4.8 mmol/L) | 1849 | - | -0.4 (-1.2; 0.5) | 0.2 (-0.3; 0.8) | 1.0 (0.2; 1.9) | 1.7 (0.5; 2.9) | 2.1 (0.7; 3.5) | 2.1 (0.8; 3.3) | 1.5 (0.0; 3.1) |
| Q5 (4.9-6.0 mmol/L) | 1374 | - | -0.8 (-1.8; 0.1) | 0.5 (-0.1; 1.2) | 2.3 (1.4; 3.2) | 3.9 (2.5; 5.2) | 4.8 (3.3; 6.3) | 4.7 (3.4; 6.1) | 3.6 (1.8; 5.3) |
| per sd increase | 8797 | - | -0.2 (-0.5; 0.1) | 0.3 (0.1; 0.5) | 0.8 (0.6; 1.1) | 1.4 (0.9; 1.8) | 1.7 (1.2; 2.1) | 1.7 (1.3; 2.1) | 1.4 (0.9; 1.9) |
| 2-h postload glucose |  |  |  |  |  |  |  |  |  |
| Q1 (≤ 4.5 mmol/L) | 1921 | - | REF | REF | REF | REF | REF | REF | REF |
| Q2 (4.6-5.1 mmol/L) | 1756 | - | -0.5 (-1.3; 0.4) | 0.1 (-0.5; 0.6) | 0.7 (-0.1; 1.5) | 1.2 (0.0; 2.4) | 1.4 (0.1; 2.7) | 1.2 (0.0; 2.5) | 0.6 (-0.9; 2.2) |
| Q3 (5.2-5.6 mmol/L) | 1615 | - | -0.5 (-1.4; 0.4) | 0.0 (-0.5; 0.6) | 0.7 (-0.1; 1.6) | 1.3 (0.0; 2.5) | 1.6 (0.2; 3.0) | 1.5 (0.2; 2.7) | 0.9 (-0.7; 2.5) |
| Q4 (5.7-6.3 mmol/L) | 1787 | - | -0.1 (-1.0; 0.7) | 0.7 (0.1; 1.2) | 1.6 (0.8; 2.4) | 2.3 (1.1; 3.6) | 2.7 (1.3; 4.0) | 2.4 (1.2; 3.6) | 1.4 (-0.2; 3.0) |
| Q5 (6.4-7.7 mmol/L) | 1706 | - | -0.2 (-1.0; 0.7) | 0.6 (0.0; 1.2) | 1.6 (0.8; 2.4) | 2.6 (1.4; 3.9) | 3.4 (2.0; 4.8) | 3.9 (2.6; 5.1) | 3.9 (2.3; 5.5) |
| per sd increase | 8785 | - | 0.0 (-0.4; 0.4) | 0.3 (0.0; 0.5) | 0.7 (0.3; 1.0) | 1.1 (0.5; 1.6) | 1.4 (0.8; 2.0) | 1.5 (1.0; 2.1) | 1.5 (0.8; 2.2) |
| **EFW (gram)** | **N** | **12 wks** | **16 wks** | **20 wks** | **24 wks** | **28 wks** | **32 wks** | **36 wks** | **40 wks** |
| Fasting glucose |  |  |  |  |  |  |  |  |  |
| Q1 (≤ 4.1 mmol/L) | 1887 | - | REF | REF | REF | REF | REF | REF | REF |
| Q2 (4.2-4.3 mmol/L) | 1907 | - | 1 (-3; 6) | 0 (-2; 3) | 1 (-6; 8) | 4 (-9; 17) | 12 (-5; 29) | 27 (8; 47) | 52 (25; 78) |
| Q3 (4.4.-4.5 mmol/L) | 1877 | - | 1 (-3; 6) | 2 (-1; 4) | 4 (-3; 12) | 11 (-2; 24) | 24 (6; 41) | 44 (25; 64) | 75 (48; 101) |
| Q4 (4.6-4.8 mmol/L) | 1880 | - | 1 (-3; 6) | 1 (-1; 4) | 4 (-3; 11) | 12 (-1; 26) | 29 (12; 47) | 58 (38; 78) | 102 (74;129) |
| Q5 (4.9-6.0 mmol/L) | 1402 | - | 2 (-3; 7) | 2 (-1; 6) | 8 (0; 16) | 25 (10; 40) | 57 (37; 77) | 111 (88; 133) | 191 (161;221) |
| per sd increase | 8953 | - | 1 (-1; 2) | 1 (0; 2) | 3 (1; 6) | 9 (5; 14) | 21 (15; 27) | 39 (33; 46) | 67 (58; 76) |
| 2-h postload glucose |  |  |  |  |  |  |  |  |  |
| Q1 (≤ 4.5 mmol/L) | 1958 | - | REF | REF | REF | REF | REF | REF | REF |
| Q2 (4.6-5.1 mmol/L) | 1790 | - | -2 (-7; 2) | 1 (-2; 4) | 6 (-1; 13) | 11 (-2; 24) | 12 (-5; 30) | 8 (-12; 27) | -6 (-33; 21) |
| Q3 (5.2-5.6 mmol/L) | 1637 | - | 1 (-3; 6) | 1 (-2; 4) | 2 (-6; 9) | 4 (-10; 18) | 9 (-9; 27) | 18 (-3; 38) | 31 (4; 58) |
| Q4 (5.7-6.3 mmol/L) | 1819 | - | 2 (-2; 7) | 4 (1; 6) | 8 (0; 15) | 16 (2; 29) | 29 (12; 47) | 50 (30; 70) | 79 (52;106) |
| Q5 (6.4-7.7 mmol/L) | 1736 | - | 2 (-3; 7) | 3 (0; 6) | 8 (1; 15) | 20 (6; 34) | 43 (25; 61) | 79 (59; 99) | 132 (104;160) |
| per sd increase | 8940 | - | 1 (-1; 2) | 1 (0; 2) | 3 (0; 5) | 7 (3; 12) | 16 (10; 22) | 30 (24; 37) | 52 (43; 61) |

Predicted differences in mean head circumference (HC), femur length (FL), abdominal circumference (AC) and estimated fetal weight (EFW) by fasting and 2-hour postload glucose levels below the diagnostic threshold for GD at 4-weekly gestational age intervals from 12/16 weeks through 40 weeks. All mean differences are estimated using multilevel fractional polynomial models with adjustment for infant sex and maternal age at delivery, ethnicity, parity, height, body mass index, smoking and alcohol use during pregnancy, education and hypertensive disorders of pregnancy. Mean differences are presented by quintiles (with the first quintile as reference group) and per standard deviation increase in fasting and 2-hour postload glucose levels.

**Table S7.** Predicted differences in mean fetal size across gestation by ethnicity and gestational diabetes – model 1.

|  |  | **Predicted mean difference (95% CI)** | | |
| --- | --- | --- | --- | --- |
| **HC (mm)** | **N** | **12 wks** | **24 wks** | **40 wks** |
| Ethnicity |  |  |  |  |
| White European | 4744 | REF | REF | REF |
| South Asian | 5956 | 0.1 (-0.4; 0.6) | -1.6 (-1.9; -1.2) | -4.7 (-5.2; -4.2) |
| GDM |  |  |  |  |
| No | 9868 | REF | REF | REF |
| Yes | 832 | -1.9 (-2.7; -1.1) | 0.8 (0.3; 1.4) | 0.1 (-0.8; 1.1) |
| Ethnicity / GDM |  |  |  |  |
| White European / no GDM | 4534 | REF | REF | REF |
| White European / GDM | 210 | -2.9 (-4.5; -1.3) | 1.9 (0.9; 3.0) | 1.4 (-0.5; 3.2) |
| South Asian / no GDM | 5334 | 0.1 (-0.4; 0.6) | -1.6 (-1.9; -1.2) | -4.7 (-5.3; -4.2) |
| South Asian / GDM | 622 | -1.5 (-2.4; -0.6) | -0.7 (-1.3; -0.0) | -3.8 (-4.8; -2.7) |
| **FL (mm)** | **N** | **12 wks** | **24 wks** | **40 wks** |
| Ethnicity |  |  |  |  |
| White European | 4740 | REF | REF | REF |
| South Asian | 5949 | 0.1 (-0.2; 0.3) | 0.1 (0.0; 0.2) | -0.1 (-0.3; 0.1) |
| GDM |  |  |  |  |
| No | 9857 | REF | REF | REF |
| Yes | 832 | -0.5 (-0.8; -0.2) | 0.2 (0.1; 0.3) | 0.4 (0.1; 0.7) |
| Ethnicity / GDM |  |  |  |  |
| White European / no GDM | 4530 | REF | REF | REF |
| White European / GDM | 210 | 0.1 (-0.5; 0.7) | 0.2 (0.0; 0.5) | 0.9 (0.4; 1.4) |
| South Asian / no GDM | 5327 | 0.2 (-0.1; 0.4) | 0.1 (0.0; 0.2) | -0.1 (-0.3; 0.1) |
| South Asian / GDM | 622 | -0.6 (-0.9; -0.2) | 0.3 (0.1; 0.4) | 0.2 (-0.2; 0.5) |
| **AC (mm)** | **N** | **16 wks** | **24 wks** | **40 wks** |
| Ethnicity |  |  |  |  |
| White European | 4648 | REF | REF | REF |
| South Asian | 5872 | -2.5 (-3.0; -2.0) | -3.9 (-4.3; -3.4) | -12.1 (-13.1; -11.2) |
| GDM |  |  |  |  |
| No | 9690 | REF | REF | REF |
| Yes | 830 | -2.4 (-3.3; -1.6) | 1.3 (0.6; 2.1) | 4.1 (2.3; 5.8) |
| Ethnicity / GDM |  |  |  |  |
| White European / no GDM | 4438 | REF | REF | REF |
| White European / GDM | 210 | -2.5 (-4.1; -1.0) | 2.0 (0.7; 3.4) | 5.9 (2.6; 9.1) |
| South Asian / no GDM | 5252 | -2.3 (-2.8; -1.8) | -4.1 (-4.6; -3.6) | -12.7 (-13.6; -11.7) |
| South Asian / GDM | 620 | -4.1 (-5.1; -3.1) | -1.9 (-2.8; -1.1) | -5.7 (-7.7; -3.7) |
| **EFW (gram)** | **N** | **16 wks** | **24 wks** | **40 wks** |
| Ethnicity |  |  |  |  |
| White European | 4745 | REF | REF | REF |
| South Asian | 5956 | 0 (-3; 2) | -24 (-28; -20) | -183 (-200; -166) |
| GDM |  |  |  |  |
| No | 9869 | REF | REF | REF |
| Yes | 832 | -8 (-12; -4) | 11 (5; 17) | 173 (140; 206) |
| Ethnicity / GDM |  |  |  |  |
| White European / no GDM | 4535 | REF | REF | REF |
| White European / GDM | 210 | -6 (-13; 2) | 16 (4; 28) | 254 (192; 316) |
| South Asian / no GDM | 5334 | 1 (-2; 4) | -26 (-31; -22) | -193 (-211; -175) |
| South Asian / GDM | 622 | -8 (-13; -3) | -10 (-17; -3) | 6 (-31; 44) |

Separate and joint associations of ethnicity and gestational diabetes with fetal growth trajectories, presented as differences in mean head circumference (HC), femur length (FL), abdominal circumference (AC) and estimated fetal weight (EFW) at three time points during gestation. All mean differences are estimated using multilevel fractional polynomial models with adjustment for infant sex only. Abbreviations: GDM = gestational diabetes; CI = confidence interval; wks = weeks.

**Table S8.** Predicted differences in mean fetal size across gestation by ethnicity and gestational diabetes– complete case analyses.

|  |  | **Predicted mean difference (95% CI)** | | |
| --- | --- | --- | --- | --- |
| **HC (mm)** | **N** | **12 wks** | **24 wks** | **40 wks** |
| Ethnicity |  |  |  |  |
| White European | 3843 | REF | REF | REF |
| South Asian | 4504 | -0.0 (-0.9; 0.8) | -1.2 (-1.7; -0.6) | -5.5 (-6.3; -4.7) |
| GDM |  |  |  |  |
| No | 7687 | REF | REF | REF |
| Yes | 660 | -1.6 (-2.5; -0.7) | 0.8 (0.2; 1.4) | 0.2 (-0.8; 1.3) |
| Ethnicity / GDM |  |  |  |  |
| White European / no GDM | 3660 | REF | REF | REF |
| White European / GDM | 183 | -2.7 (-4.4; -0.9) | 1.2 (0.1; 2.4) | -0.2 (-2.1; 1.7) |
| South Asian / no GDM | 4027 | -0.2 (-1.1; 0.7) | -1.1 (-1.7; -0.5) | -5.5 (-6.4; -4.7) |
| South Asian / GDM | 477 | -1.5 (-2.7; -0.2) | -0.5 (-1.4; 0.3) | -5.1 (-6.5; -3.8) |
| **FL (mm)** | **N** | **12 wks** | **24 wks** | **40 wks** |
| Ethnicity |  |  |  |  |
| White European | 3839 | REF | REF | REF |
| South Asian | 4501 | 0.1 (-0.3; 0.5) | 0.2 (0.1; 0.4) | 0.0 (-0.4; 0.4) |
| GDM |  |  |  |  |
| No | 7680 | REF | REF | REF |
| Yes | 660 | -0.7 (-1.1; -0.3) | 0.1 (0.0; 0.3) | 0.1 (-0.2; 0.4) |
| Ethnicity / GDM |  |  |  |  |
| White European / no GDM | 3656 | REF | REF | REF |
| White European / GDM | 183 | 0.0 (-0.7; 0.7) | 0.1 (-0.2; 0.4) | 0.5 (0.0; 1.1) |
| South Asian / no GDM | 4024 | 0.2 (-0.2; 0.6) | 0.2 (0.1; 0.4) | 0.1 (-0.3; 0.5) |
| South Asian / GDM | 477 | -0.8 (-1.3; -0.2) | 0.3 (0.1; 0.6) | 0.0 (-0.5; 0.5) |
| **AC (mm)** | **N** | **16 wks** | **24 wks** | **40 wks** |
| Ethnicity |  |  |  |  |
| White European | 3768 | REF | REF | REF |
| South Asian | 4447 | -1.8 (-2.6; -0.9) | -3.5 (-4.3; -2.7) | -13.0 (-14.6; -11.4) |
| GDM |  |  |  |  |
| No | 7556 | REF | REF | REF |
| Yes | 659 | -1.5 (-2.4; -0.6) | 0.9 (0.1; 1.7) | 4.6 (2.7; 6.4) |
| Ethnicity / GDM |  |  |  |  |
| White European / no GDM | 3585 | REF | REF | REF |
| White European / GDM | 183 | -2.2 (-3.8; -0.5) | 0.3 (-1.1; 1.8) | 4.7 (1.2; 8.1) |
| South Asian / no GDM | 3971 | -1.8 (-2.7; -1.0) | -3.5 (-4.3; -2.7) | -13.0 (-14.7; -11.4) |
| South Asian / GDM | 476 | -3.1 (-4.3; -1.8) | -2.4 (-3.6; -1.2) | -8.5 (-11.0; -5.9) |
| **EFW (gram)** | **N** | **16 wks** | **24 wks** | **40 wks** |
| Ethnicity |  |  |  |  |
| White European | 3844 | REF | REF | REF |
| South Asian | 4505 | 1 (-3; 5) | -20 (-27; -13) | -224 (-252; -196) |
| GDM |  |  |  |  |
| No | 7689 | REF | REF | REF |
| Yes | 660 | -4 (-9; 0) | 6 (-1; 13) | 171 (137; 205) |
| Ethnicity / GDM |  |  |  |  |
| White European / no GDM | 3661 | REF | REF | REF |
| White European / GDM | 183 | -4 (-12; 4) | 3 (-9; 15) | 181 (118; 243) |
| South Asian / no GDM | 4028 | 1 (-3; 6) | -20 (-27; -13) | -223 (-251; -194) |
| South Asian / GDM | 477 | -3 (-10; 3) | -12 (-22; -3) | -56 (-102; -10) |

Seperate and joint associations of ethnicity and gestational diabetes with fetal growth trajectories, presented as differences in mean head circumference (HC), femur length (FL), abdominal circumference (AC) and estimated fetal weight (EFW) at three time points during gestation. Analyses are conducted in singletons with complete covariate data. All mean differences are estimated using multilevel fractional polynomial models with adjustment for infant sex and maternal age at delivery, parity, height, body mass index, education, smoking and alcohol use during pregnancy, and hypertensive disorders of pregnancy. Models are additionally adjusted for gestational diabetes [in analyses examining mean differences by ethnicity (White European vs. South Asian)] and ethnicity [in analyses examining mean differences by gestational diabetes (yes vs. no)]. Abbreviations: GDM = gestational diabetes; CI = confidence interval; wks = weeks.

**Table S9.** Predicted differences in mean fetal size across gestation by ethnicity and gestational diabetes - analyses restricted to singletons of women with ≤ 4 repeat ultrasound scans.

|  |  | **Predicted mean difference (95% CI)** | | |
| --- | --- | --- | --- | --- |
| **HC (mm)** | **N** | **12 wks** | **24 wks** | **40 wks** |
| Ethnicity |  |  |  |  |
| White European | 4449 | REF | REF | REF |
| South Asian | 5422 | 0.1 (-0.8; 0.9) | -0.7 (-1.3; -0.2) | -5.5 (-6.3; -4.7) |
| GDM |  |  |  |  |
| No | 9375 | REF | REF | REF |
| Yes | 496 | -1.4 (-2.6; -0.2) | 1.0 (0.3; 1.7) | -0.7 (-1.9; 0.5) |
| Ethnicity / GDM |  |  |  |  |
| White European / no GDM | 4328 | REF | REF | REF |
| White European / GDM | 121 | -3.0 (-5.5; -0.5) | 1.1 (-0.2; 2.5) | -0.9 (-3.2; 1.5) |
| South Asian / no GDM | 5047 | 0.0 (-0.9; 0.8) | -0.7 (-1.3; -0.2) | -5.4 (-6.2; -4.7) |
| South Asian / GDM | 375 | -0.9 (-2.5; 0.7) | 0.2 (-0.7; 1.1) | -6.1 (-7.7; -4.6) |
| **FL (mm)** | **N** | **12 wks** | **24 wks** | **40 wks** |
| Ethnicity |  |  |  |  |
| White European | 4445 | REF | REF | REF |
| South Asian | 5415 | 0.1 (-0.3; 0.5) | 0.3 (0.1; 0.5) | 0.0 (-0.4; 0.4) |
| GDM |  |  |  |  |
| No | 9364 | REF | REF | REF |
| Yes | 496 | -0.3 (-0.8; 0.2) | 0.1 (-0.1; 0.3) | -0.1 (-0.5; 0.3) |
| Ethnicity / GDM |  |  |  |  |
| White European / no GDM | 4324 | REF | REF | REF |
| White European / GDM | 121 | 0.0 (-1.0; 1.1) | 0.1 (-0.3; 0.4) | 0.1 (-0.5; 0.8) |
| South Asian / no GDM | 5040 | 0.2 (-0.2; 0.5) | 0.3 (0.1; 0.5) | 0.0 (-0.4; 0.4) |
| South Asian / GDM | 375 | -0.3 (-0.9; 0.4) | 0.4 (0.2; 0.7) | -0.2 (-0.7; 0.3) |
| **AC (mm)** | **N** | **16 wks** | **24 wks** | **40 wks** |
| Ethnicity |  |  |  |  |
| White European | 4353 | REF | REF | REF |
| South Asian | 5338 | -1.8 (-2.6; -1.0) | -2.9 (-3.8; -2.1) | -12.6 (-14.1; -11.0) |
| GDM |  |  |  |  |
| No | 9197 | REF | REF | REF |
| Yes | 494 | -1.5 (-2.6; -0.4) | 0.8 (-0.1; 1.8) | 1.9 (-0.2; 4.0) |
| Ethnicity / GDM |  |  |  |  |
| White European / no GDM | 4232 | REF | REF | REF |
| White European / GDM | 121 | -1.9 (-3.9; 0.2) | -0.1 (-1.9; 1.7) | 0.8 (-3.4; 5.0) |
| South Asian / no GDM | 4965 | -1.8 (-2.6; -1.0) | -3.0 (-3.8; -2.2) | -12.6 (-14.2; -11.1) |
| South Asian / GDM | 373 | -3.2 (-4.6; -1.8) | -1.8 (-3.1; -0.6) | -10.4 (-13.1; -7.6) |
| **EFW (gram)** | **N** | **16 wks** | **24 wks** | **40 wks** |
| Ethnicity |  |  |  |  |
| White European | 4450 | REF | REF | REF |
| South Asian | 5422 | 1 (-4; 5) | -15 (-23; -8) | -200 (-226; -174) |
| GDM |  |  |  |  |
| No | 9376 | REF | REF | REF |
| Yes | 496 | -4 (-10; 1) | 9 (1; 17) | 116 (78; 154) |
| Ethnicity / GDM |  |  |  |  |
| White European / no GDM | 4329 | REF | REF | REF |
| White European / GDM | 121 | -2 (-12; 8) | 1 (-15; 17) | 101 (25; 177) |
| South Asian / no GDM | 5047 | 1 (-3; 6) | -16 (-24; -9) | -201 (-227; -175) |
| South Asian / GDM | 375 | -4 (-11; 3) | -4 (-15; 7) | -80 (-129; -31) |

Separate and joint associations of ethnicity and gestational diabetes with fetal growth trajectories, presented as mean differences of head circumference (HC), femur length (FL), abdominal circumference (AC) and estimated fetal weight (EFW) at three time points during gestation. Analyses are restricted with to singletons of women with ≤ 4 repeat ultrasound scans. All mean differences are estimated using multilevel fractional polynomial models with adjustment for infant sex and maternal age at delivery, parity, height, body mass index, education, smoking and alcohol use during pregnancy, and hypertensive disorders of pregnancy. Models are additionally adjusted for gestational diabetes [in analyses examining mean differences by ethnicity (White European vs. South Asian)] and ethnicity [in analyses examining mean differences by gestational diabetes (yes vs. no)]. Abbreviations: GDM = gestational diabetes; CI = confidence interval; wks = weeks.

**Table S10.** Predicted differences in mean fetal size across gestation by ethnicity and gestational diabetes – analyses restricted to singletons of women who had their OGTT between 24 and 30 weeks.

|  |  | **Predicted mean difference (95% CI)** | | |
| --- | --- | --- | --- | --- |
| **HC (mm)** | **N** | **12 wks** | **24 wks** | **40 wks** |
| Ethnicity |  |  |  |  |
| White European | 4064 | REF | REF | REF |
| South Asian | 5109 | 0.3 (-0.5; 1.1) | -1.1 (-1.7; -0.6) | -5.7 (-6.5; -4.8) |
| GDM |  |  |  |  |
| No | 8464 | REF | REF | REF |
| Yes | 709 | -1.4 (-2.2; -0.6) | 1.1 (0.5 - 1.6) | 0.0 (-1.0; 1.0) |
| Ethnicity / GDM |  |  |  |  |
| White European / no GDM | 3880 | REF | REF | REF |
| White European / GDM | 184 | -2.3 (-3.9; -0.7) | 1.9 (0.8; 3.0) | 0.2 (-1.7; 2.1) |
| South Asian / no GDM | 4584 | 0.2 (-0.6; 1.0) | -1.0 (-1.6; -0.5) | -5.6 (-6.5; -4.8) |
| South Asian / GDM | 525 | -1.0 (-2.1; 0.1) | -0.3 (-1.1; 0.5) | -5.7 (-7.1; -4.4) |
| **FL (mm)** | **N** | **12 wks** | **24 wks** | **40 wks** |
| Ethnicity |  |  |  |  |
| White European | 4061 | REF | REF | REF |
| South Asian | 5105 | 0.1 (-0.3; 0.4) | 0.3 (0.1; 0.4) | -0.2 (-0.6; 0.2) |
| GDM |  |  |  |  |
| No | 8457 | REF | REF | REF |
| Yes | 709 | -0.6 (-0.9; -0.2) | 0.1 (-0.1; 0.2) | 0.2 (-0.1; 0.5) |
| Ethnicity / GDM |  |  |  |  |
| White European / no GDM | 3877 | REF | REF | REF |
| White European / GDM | 184 | 0.2 (-0.5; 0.9) | 0.1 (-0.2; 0.3) | 0.6 (0.0; 1.1) |
| South Asian / no GDM | 4580 | 0.2 (-0.2; 0.5) | 0.3 (0.1; 0.4) | -0.1 (-0.5; 0.3) |
| South Asian / GDM | 525 | -0.7 (-1.2; -0.2) | 0.4 (0.1; 0.6) | -0.1 (-0.6; 0.4) |
| **AC (mm)** | **N** | **16 wks** | **24 wks** | **40 wks** |
| Ethnicity |  |  |  |  |
| White European | 3981 | REF | REF | REF |
| South Asian | 5046 | -1.7 (-2.5; -0.8) | -3.5 (-4.3; -2.7) | -12.6 (-14.2; -11.1) |
| GDM |  |  |  |  |
| No | 8320 | REF | REF | REF |
| Yes | 707 | -1.7 (-2.6; -0.8) | 0.9 (0.1; 1.7) | 4.9 (3.1; 6.7) |
| Ethnicity / GDM |  |  |  |  |
| White European / no GDM | 3797 | REF | REF | REF |
| White European / GDM | 184 | -2.3 (-3.9; -0.7) | 1.0 (-0.4; 2.5) | 3.8 (0.4; 7.2) |
| South Asian / no GDM | 4523 | -1.7 (-2.6; -0.9) | -3.5 (-4.3; -2.7) | -12.8 (-14.4; -11.1) |
| South Asian / GDM | 523 | -3.2 (-4.4; -1.9) | -2.6 (-3.7; -1.5) | -7.4 (-9.8; -5.0) |
| **EFW (gram)** | **N** | **16 wks** | **24 wks** | **40 wks** |
| Ethnicity |  |  |  |  |
| White European | 4065 | REF | REF | REF |
| South Asian | 5109 | 2 (-3; 6) | -19 (-26; -12) | -220 (-247; -192) |
| GDM |  |  |  |  |
| No | 8465 | REF | REF | REF |
| Yes | 709 | -6 (-11; -2) | 8 (2; 15) | 159 (126; 192) |
| Ethnicity / GDM |  |  |  |  |
| White European / no GDM | 3881 | REF | REF | REF |
| White European / GDM | 184 | -4 (-12; 4) | 8 (-4; 20) | 183 (121; 246) |
| South Asian / no GDM | 4584 | 2 (-3; 6) | -19 (-26; -12) | -217 (-245; -189) |
| South Asian / GDM | 525 | -6 (-12; 1) | -10 (-20; -1) | -68 (-113; -24) |

Separate and joint associations of ethnicity and gestational diabetes with fetal growth trajectories, presented as mean differences of head circumference (HC), femur length (FL), abdominal circumference (AC) and estimated fetal weight (EFW) at three time points during gestation. Analyses are restricted to singletons of women who had their OGTT between 24 and 30 weeks of gestation. All mean differences are estimated using multilevel fractional polynomial models with adjustment for infant sex and maternal age at delivery, parity, height, body mass index, education, smoking and alcohol use during pregnancy, and hypertensive disorders of pregnancy. Models are additionally adjusted for gestational diabetes [in analyses examining mean differences by ethnicity (White European vs. South Asian)] and ethnicity [in analyses examining mean differences by gestational diabetes (yes vs. no)]. Abbreviations: GDM = gestational diabetes; CI = confidence interval; wks = weeks.

**Figure S1.** Flow chart of the study population.

**
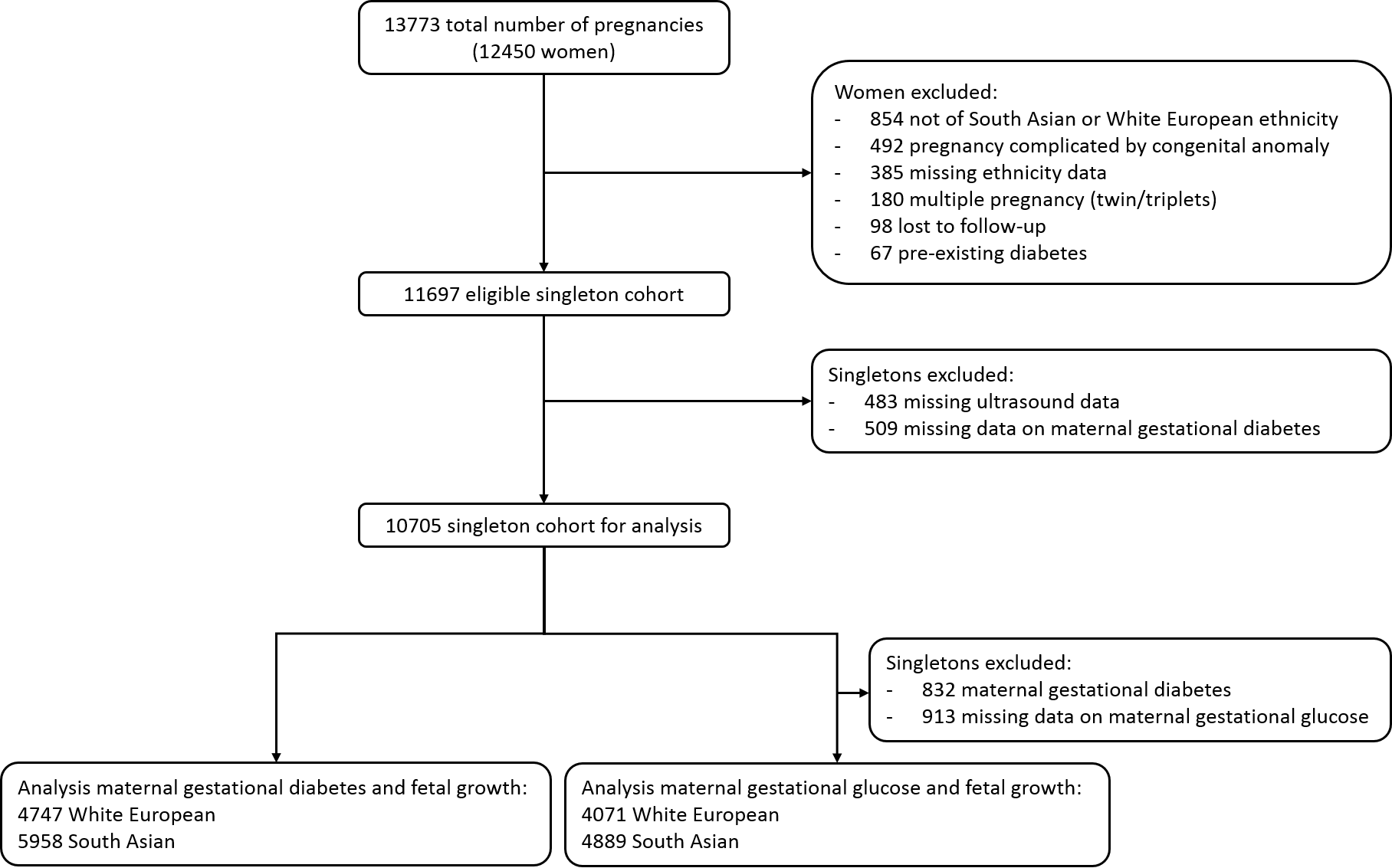
**

Pregnancies complicated by gestational diabetes (based on fasting glucose ≥ 6.1 mmol/L or 2-hour postload glucose ≥ 7.8 mmol/L) were excluded from fasting glucose and 2-hour postload glucose analyses. Information on gestational glucose was available for most pregnancies (4071 White European and 4889 South Asian) not complicated by gestational diabetes.

**Figure S2.** Best-fitting growth trajectories of each fetal parameter identified by multilevel fractional polynomial models.


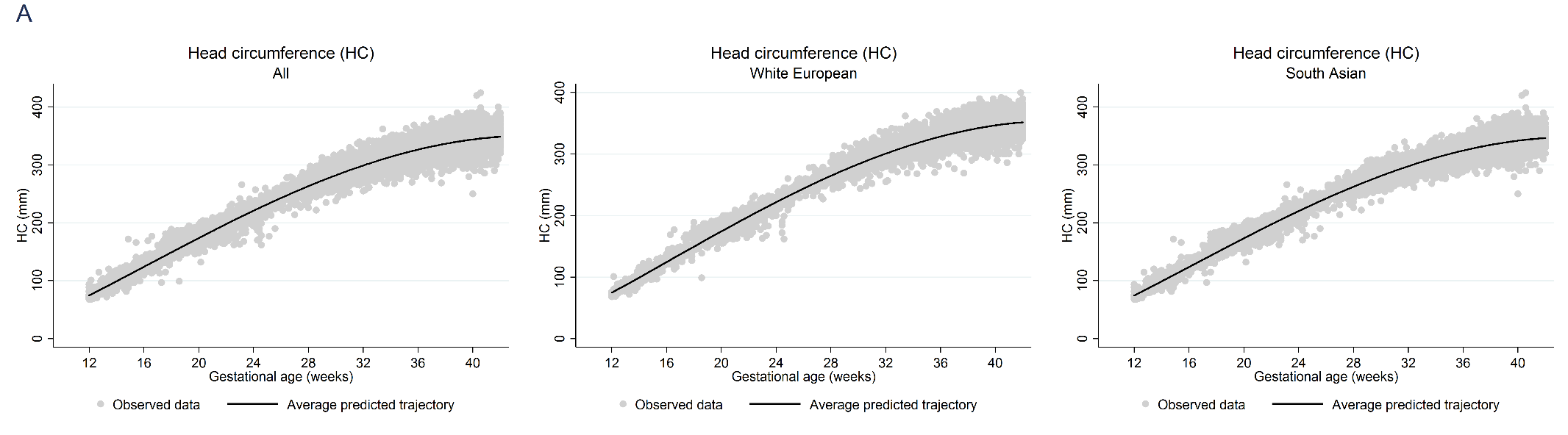


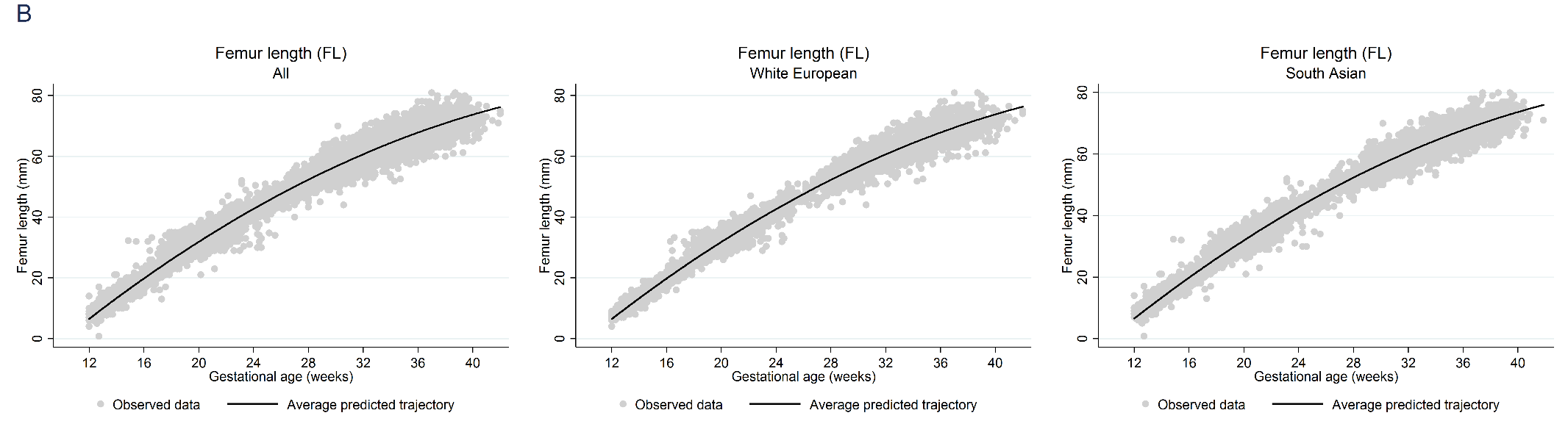


**Figure S2.** *Continued*


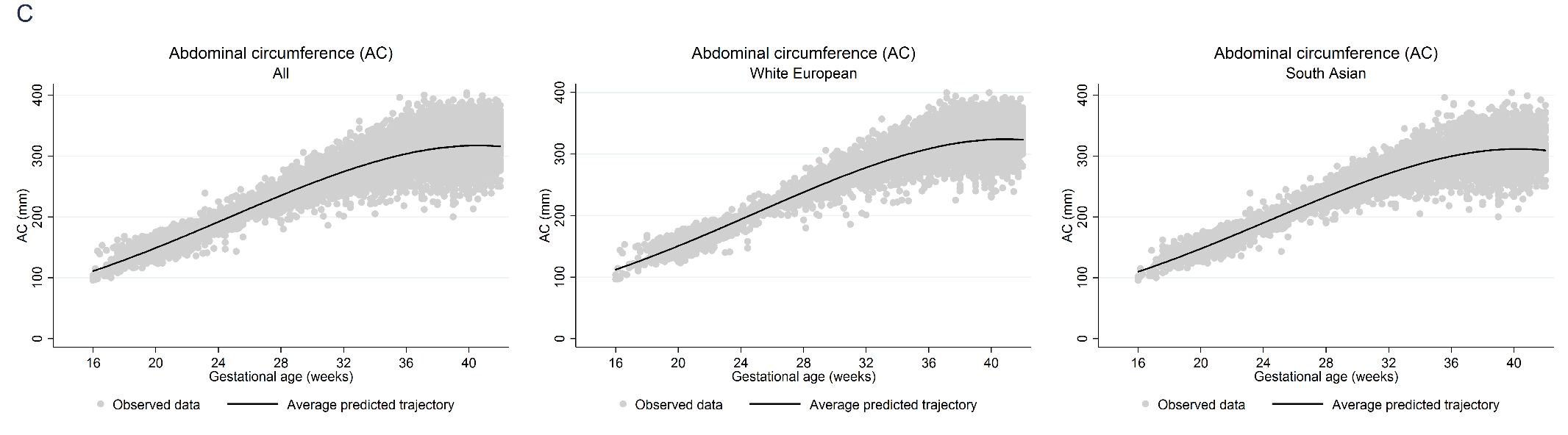


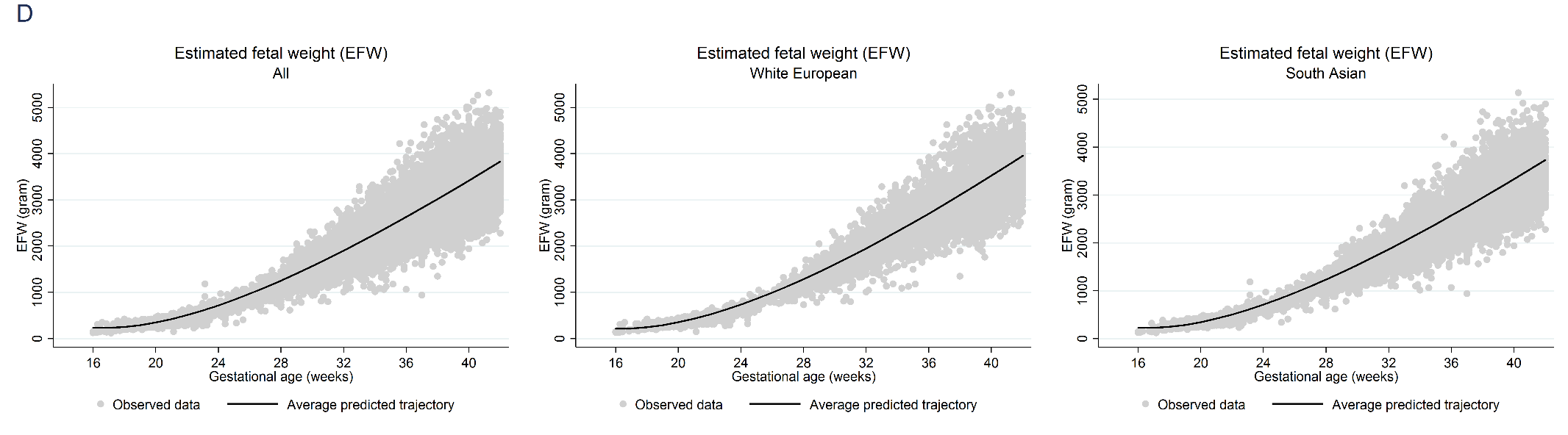


Average growth trajectories of fetal head circumference (HC), femur length (FL), abdominal circumference (AC) and estimated fetal weight (EFW) from 12/16 weeks through birth predicted by best-fitting multilevel fractional polynomial models in the total study population and in each ethnic group.

**Figure S3.** Average predicted fetal growth trajectories, stratified by ethnicity.

**A**

**
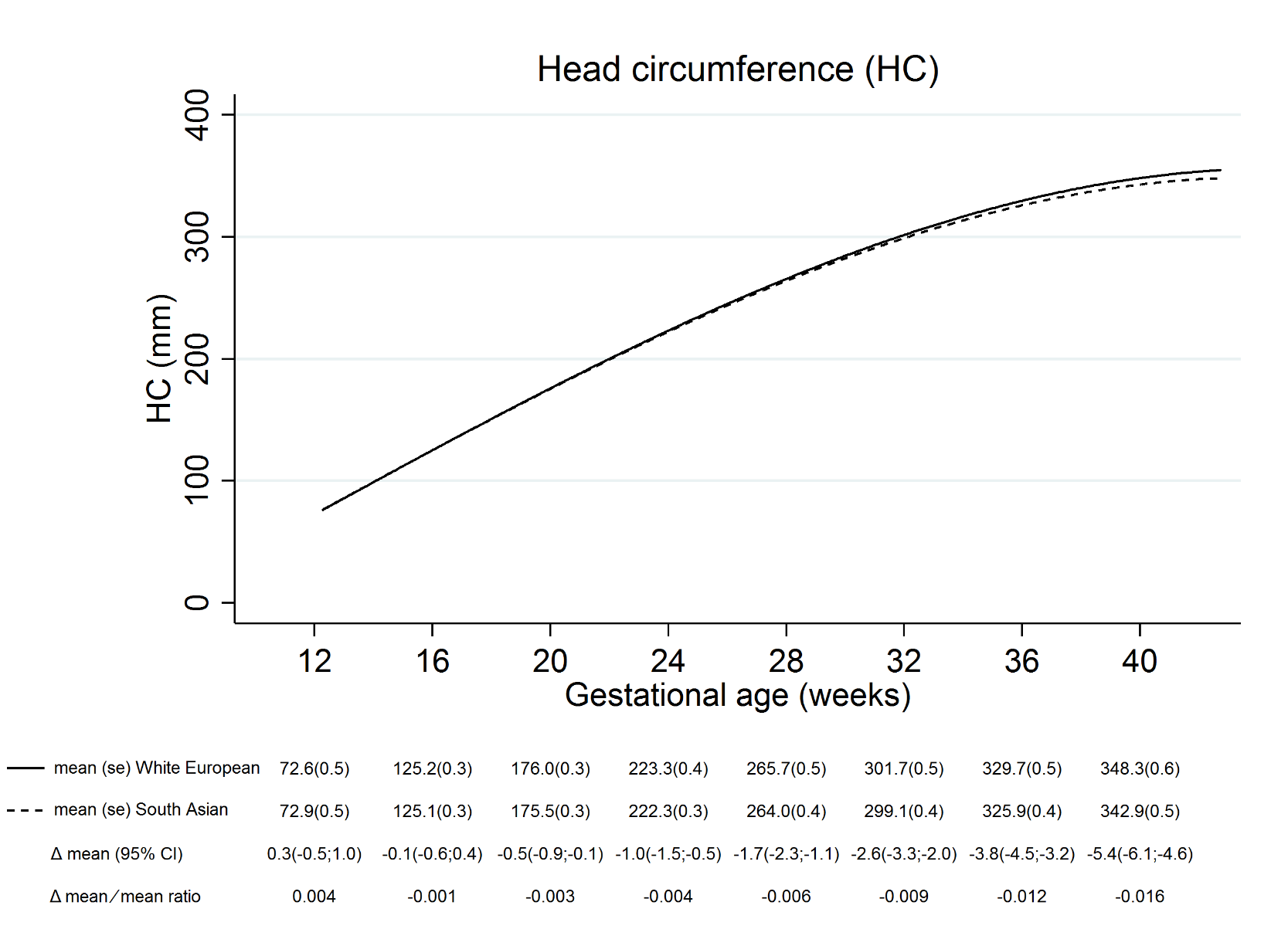
**

**B**

**
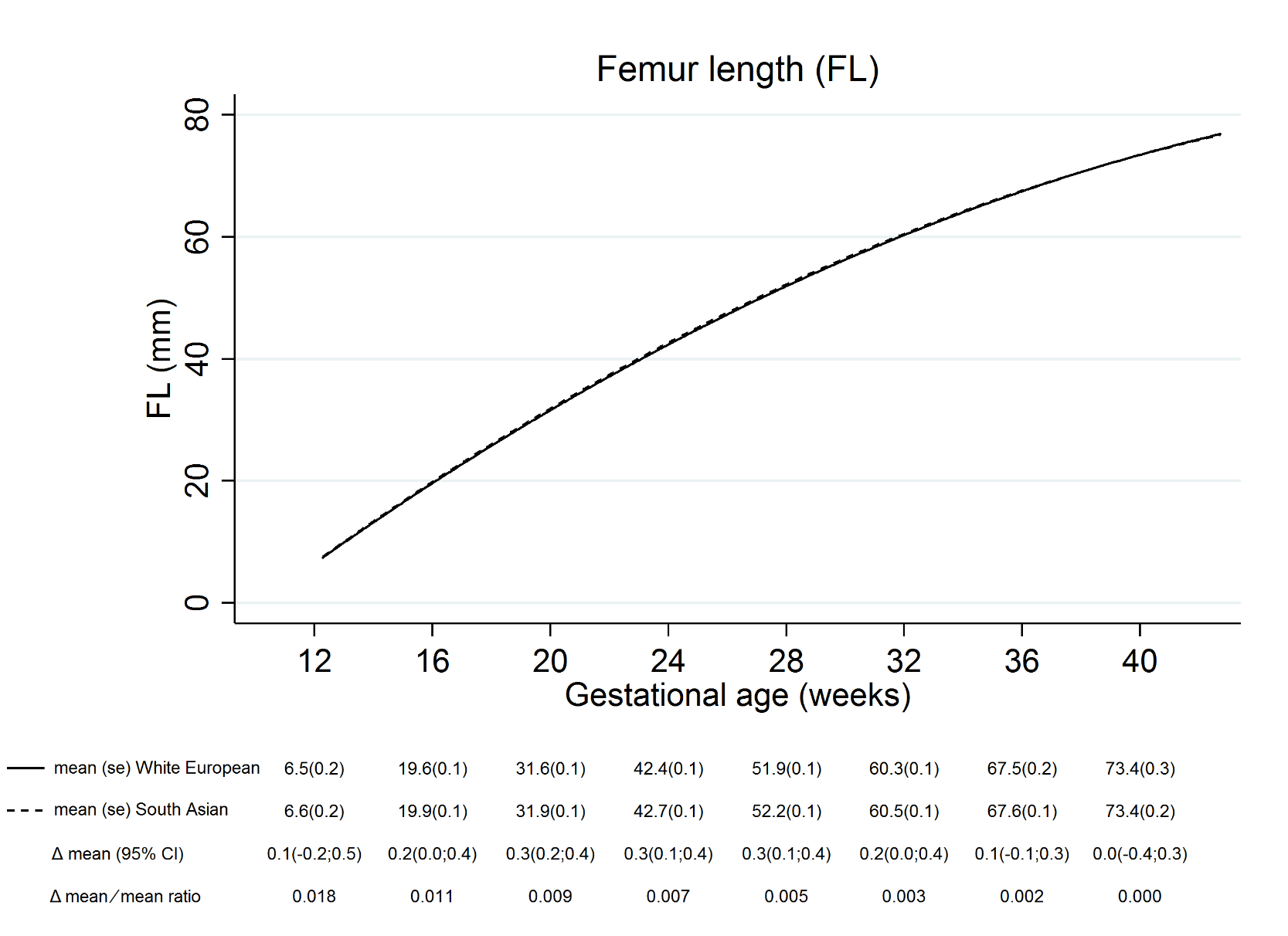
**

**C**

**
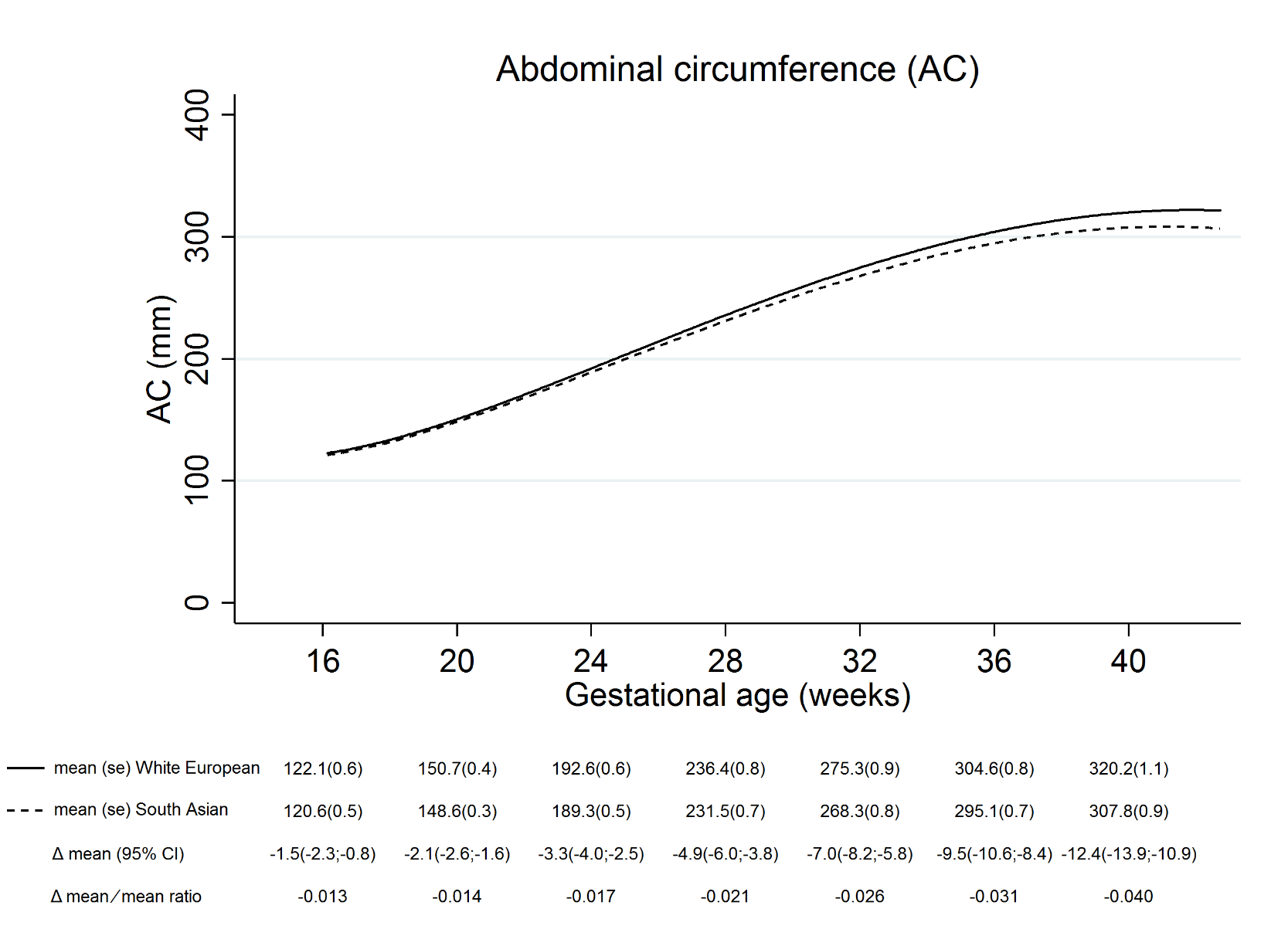
**

**D**

**
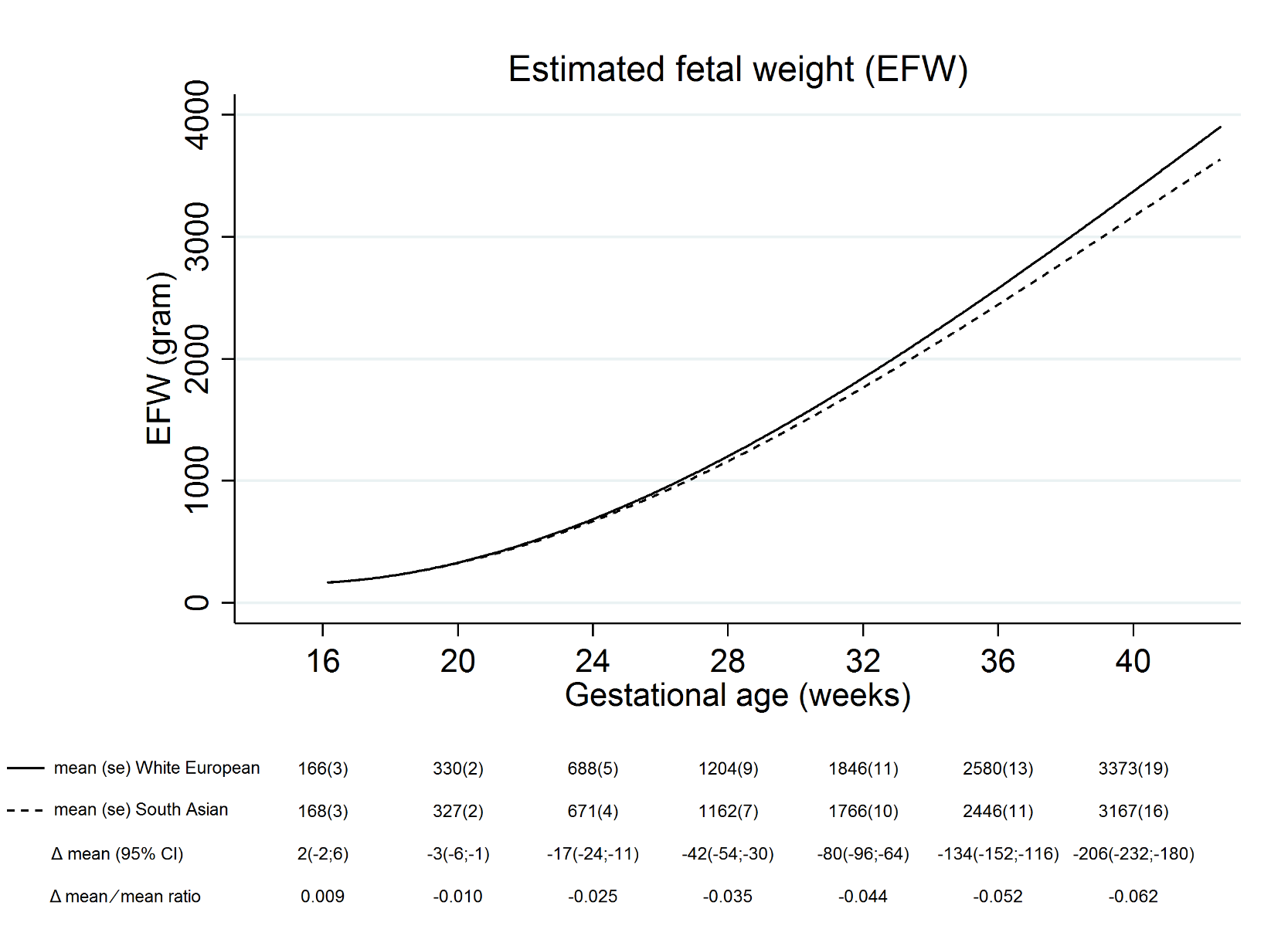
**

Average predicted growth trajectories of fetal head circumference (*A*), femur length (*B*), abdominal circumference (*C*) and estimated fetal weight (*D*) stratified by ethnic origin (South Asian vs. White European). All growth trajectories are estimated using multilevel fractional polynomial models with adjustment for infant sex and maternal age at delivery, parity, height, body mass index, education, smoking and alcohol use during pregnancy, gestational diabetes and hypertensive disorders of pregnancy. In these analyses, all covariates were set to the reference category: infant sex (male), maternal age at delivery (25-29 years), parity (nulliparous), height (160-165 cm), education level (5 GSCEs), body mass index (18.5-25 kg/m^2^), smoking during pregnancy (no), alcohol during pregnancy (no), hypertensive disorders of pregnancy (no) and gestational diabetes (no). Predicted means by ethnicity are tabulated below at 4-weekly gestational age intervals from 12/16 weeks to 40 weeks, as well as absolute mean differences (∆ mean) and proportional mean differences (∆ mean/mean ratio).

**Figure S4.** Average predicted fetal growth trajectories, stratified by gestational diabetes.

**A**

**
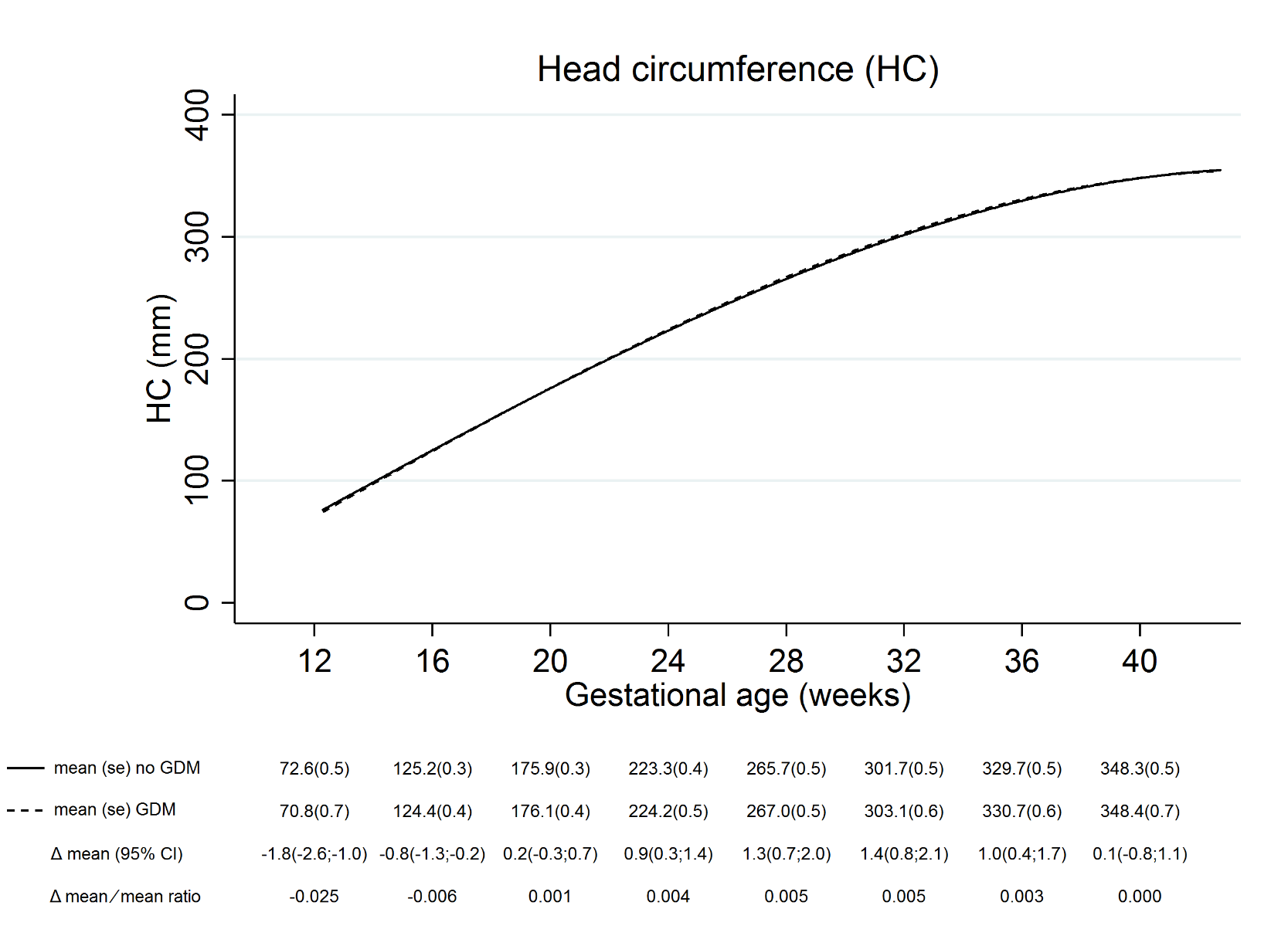
**

**B**


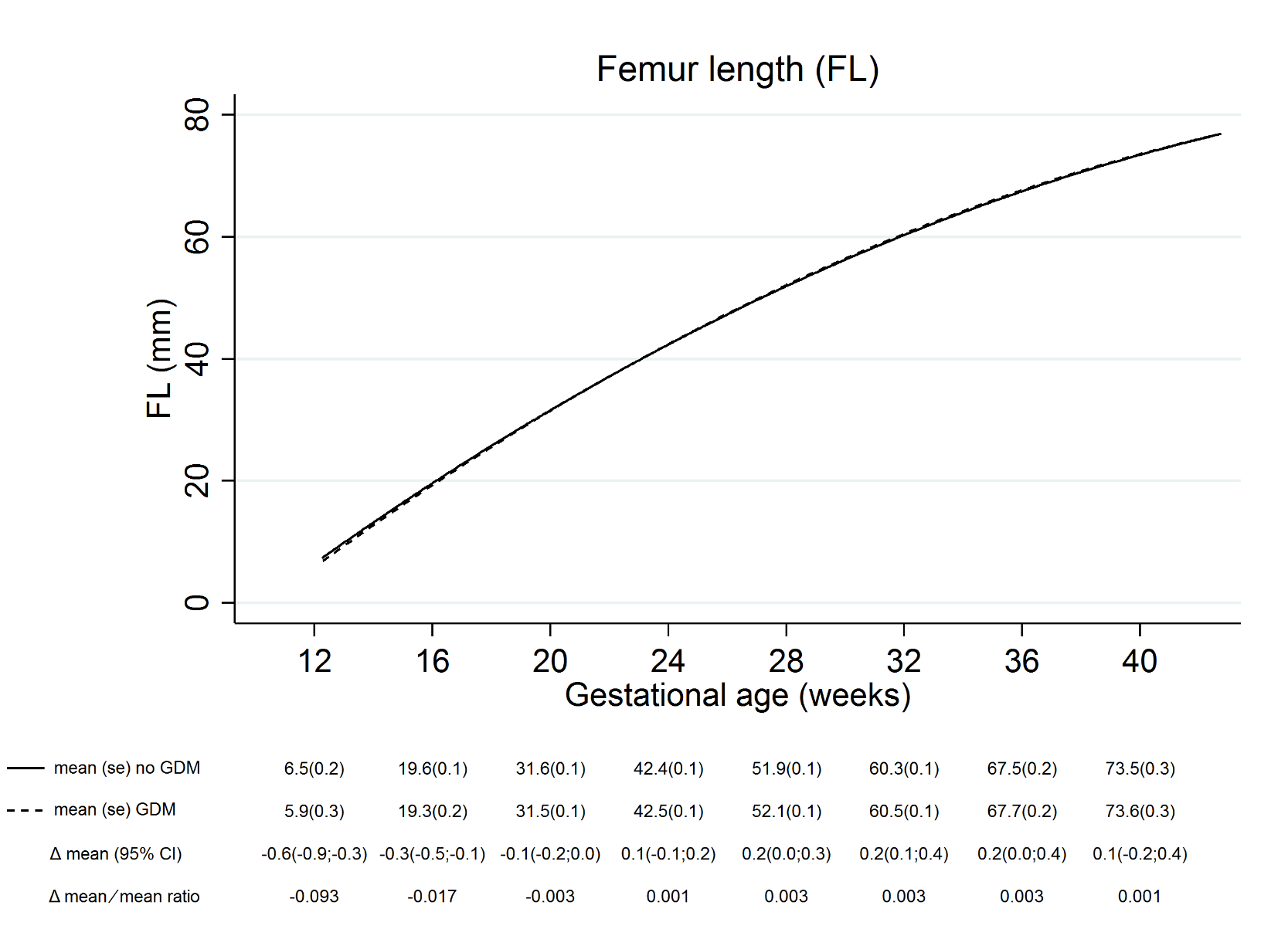


**C**

**
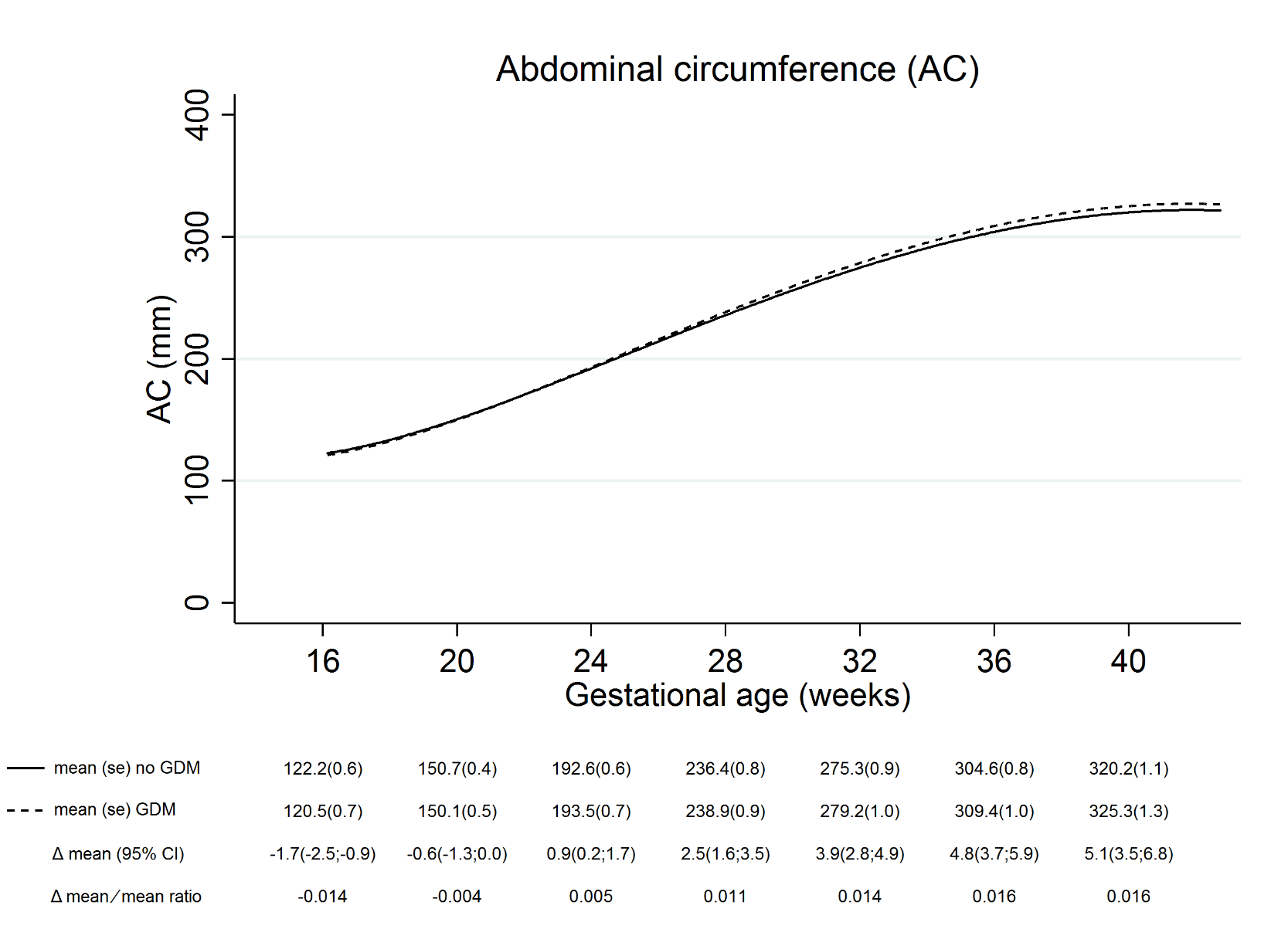
**

**D**


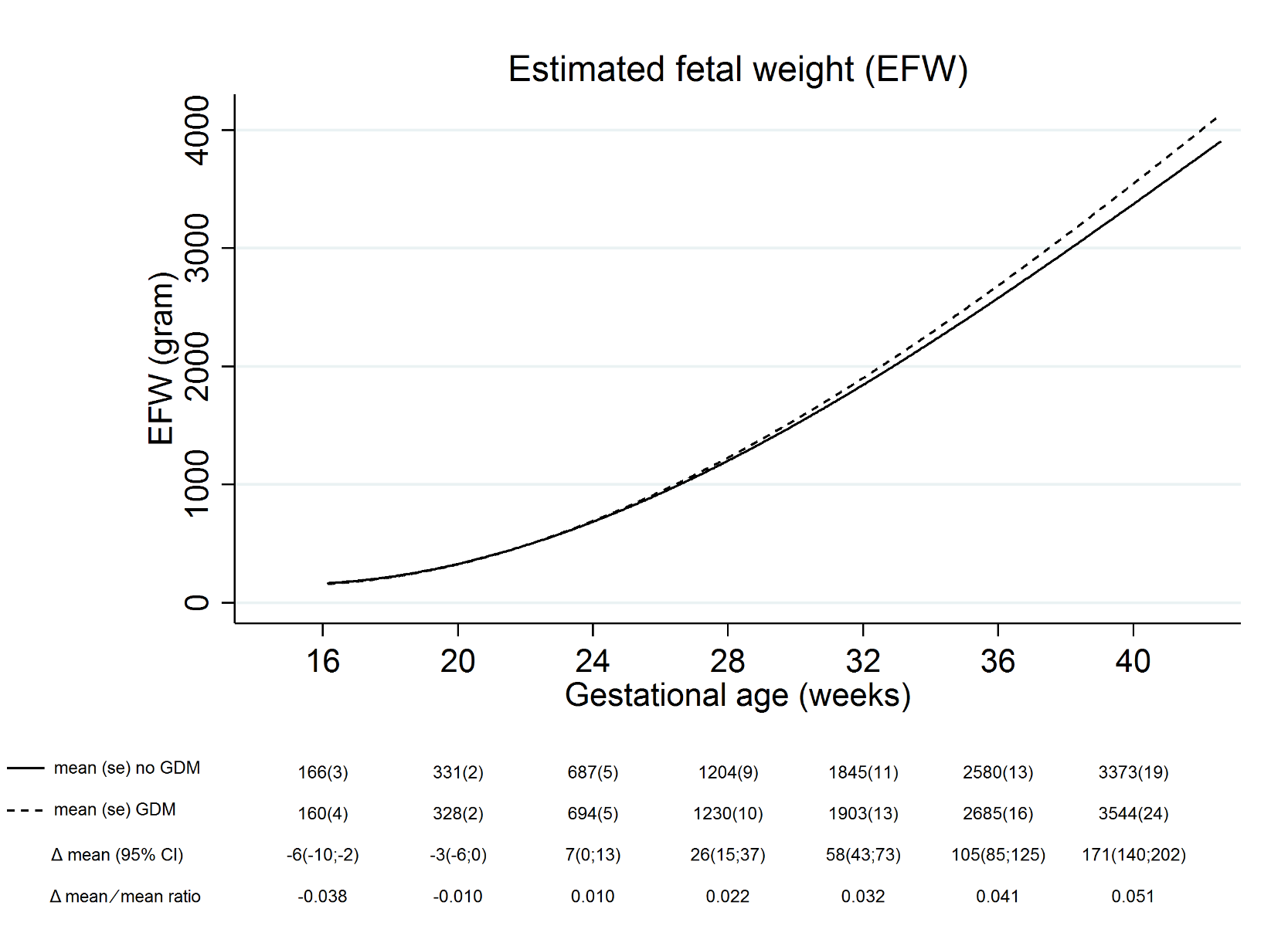


Average predicted growth trajectories of fetal head circumference (*A*), femur length (*B*), abdominal circumference (*C*) and estimated fetal weight (*D*) stratified by gestational diabetes (yes vs. no). All growth trajectories are estimated using multilevel fractional polynomial models with adjustment for infant sex and maternal age at delivery, ethnicity parity, height, body mass index, education, smoking and alcohol use during pregnancy, and hypertensive disorders of pregnancy. In these analyses, all covariates were set to the reference category: infant sex (male), maternal age at delivery (25-29 years), ethnicity (White European), parity (nulliparous), height (160-165 cm), education level (5 GSCEs), body mass index (18.5-25 kg/m^2^), smoking during pregnancy (no), alcohol during pregnancy (no), hypertensive disorders of pregnancy (no). Predicted means by gestational diabetes are tabulated below at 4-weekly gestational age intervals from 12/16 weeks to 40 weeks, as well as absolute mean differences (∆ mean) and proportional mean differences (∆ mean/mean ratio). Abbrevations: GDM = gestational diabetes.

**Figure S5.** Associations of gestational fasting glucose with fetal size across gestation in White Europeans and South Asians.


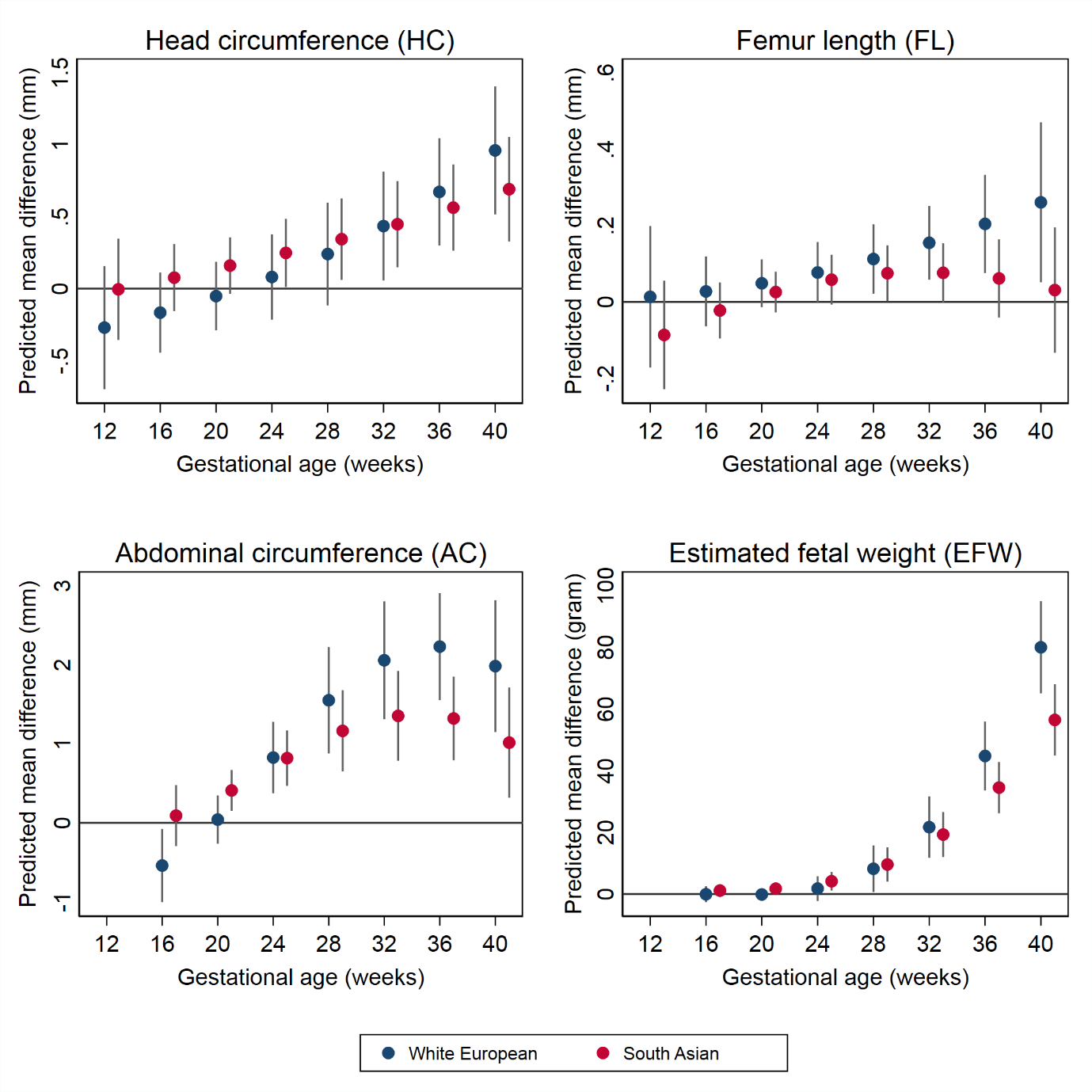


Predicted differences of mean fetal head circumference (mm), femur length (mm), abdominal circumference (mm) and estimated fetal weight (gram) per standard deviation increase in fasing glucose levels below the diagnostic threshold for gestational diabetes at 4 weekly gestational age intervals from 12/16 weeks through 40 weeks. Predicted mean differences are plotted for each ethnic group (White European vs. South Asian). All estimates are derived from multivariable models with adjustment for infant sex, maternal age at delivery, parity, height, body mass index, education level, smoking and alcohol use during pregnancy, and hypertensive disorders of pregnancy.

**Figure S6.** Associations of gestational 2-hour postload glucose with fetal size across gestation in White Europeans and South Asians.


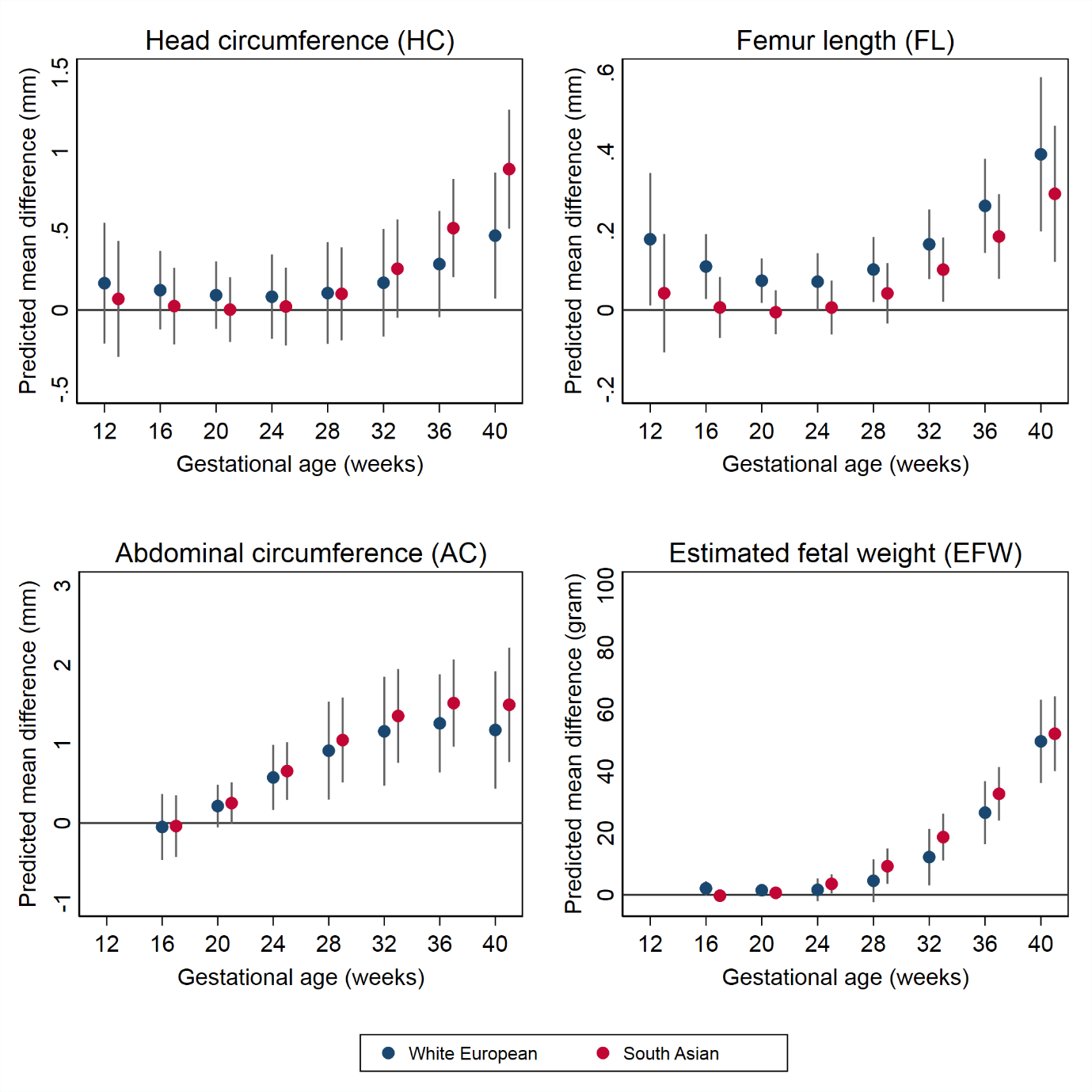


Predicted differences in mean fetal head circumference (mm), femur length (mm), abdominal circumference (mm) and estimated fetal weight (gram) per standard deviation increase in 2-hour postload glucose levels below the diagnostic threshold for gestational diabetes at 4-weekly gestational age intervals from 12/16 weeks through 40 weeks. Predicted mean differences are plotted for each ethnic group (White European and South Asian). All estimates are derived from multivariable models with adjustment for infant sex, maternal age at delivery, parity, height, body mass index, education, smoking and alcohol use during pregnancy, and hypertensive disorders of pregnancy.

**Figure S7.** Average predicted fetal growth trajectories, stratified by ethnicity and gestational diabetes.

**A**

**
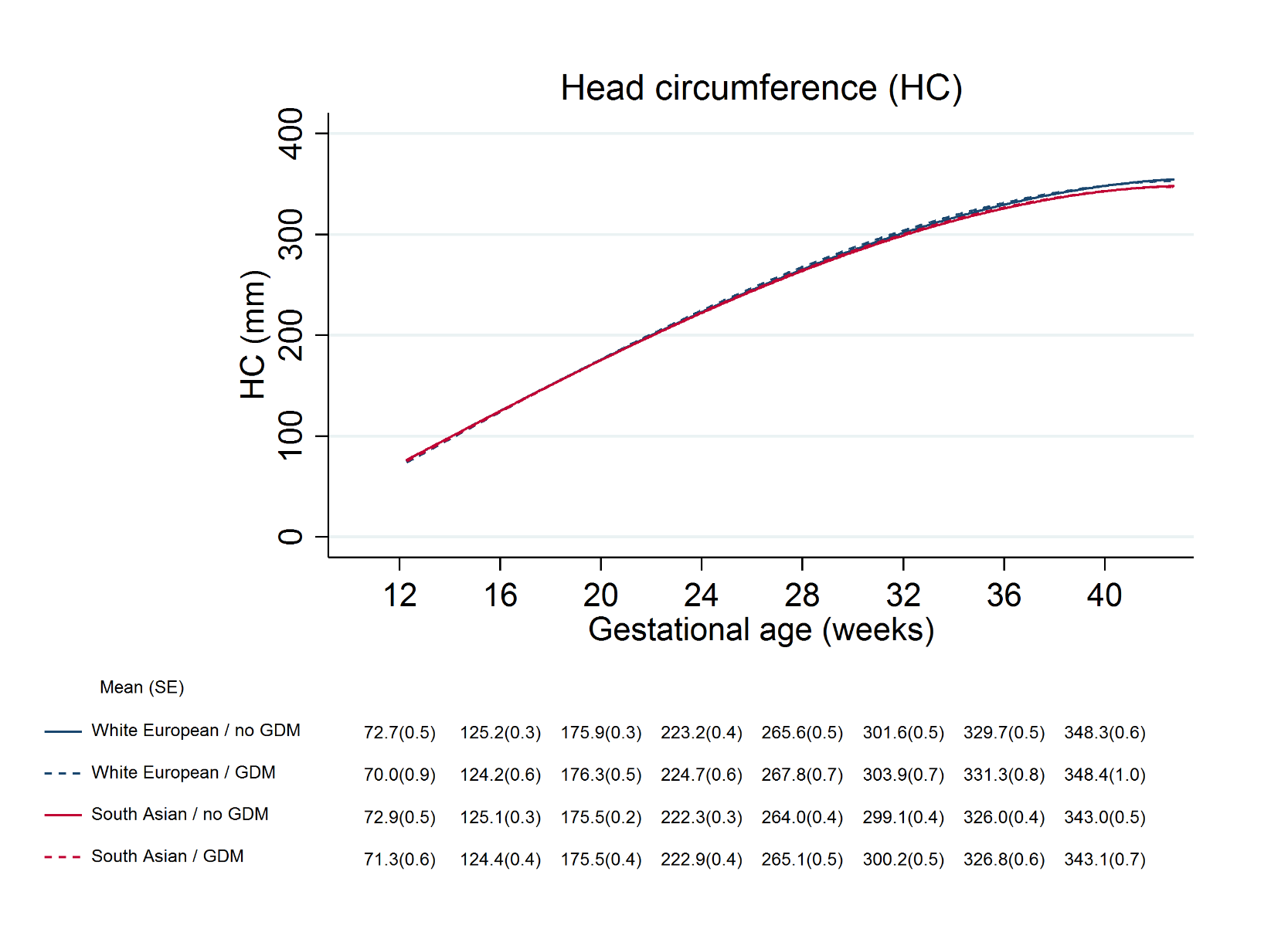
**

**B**

**
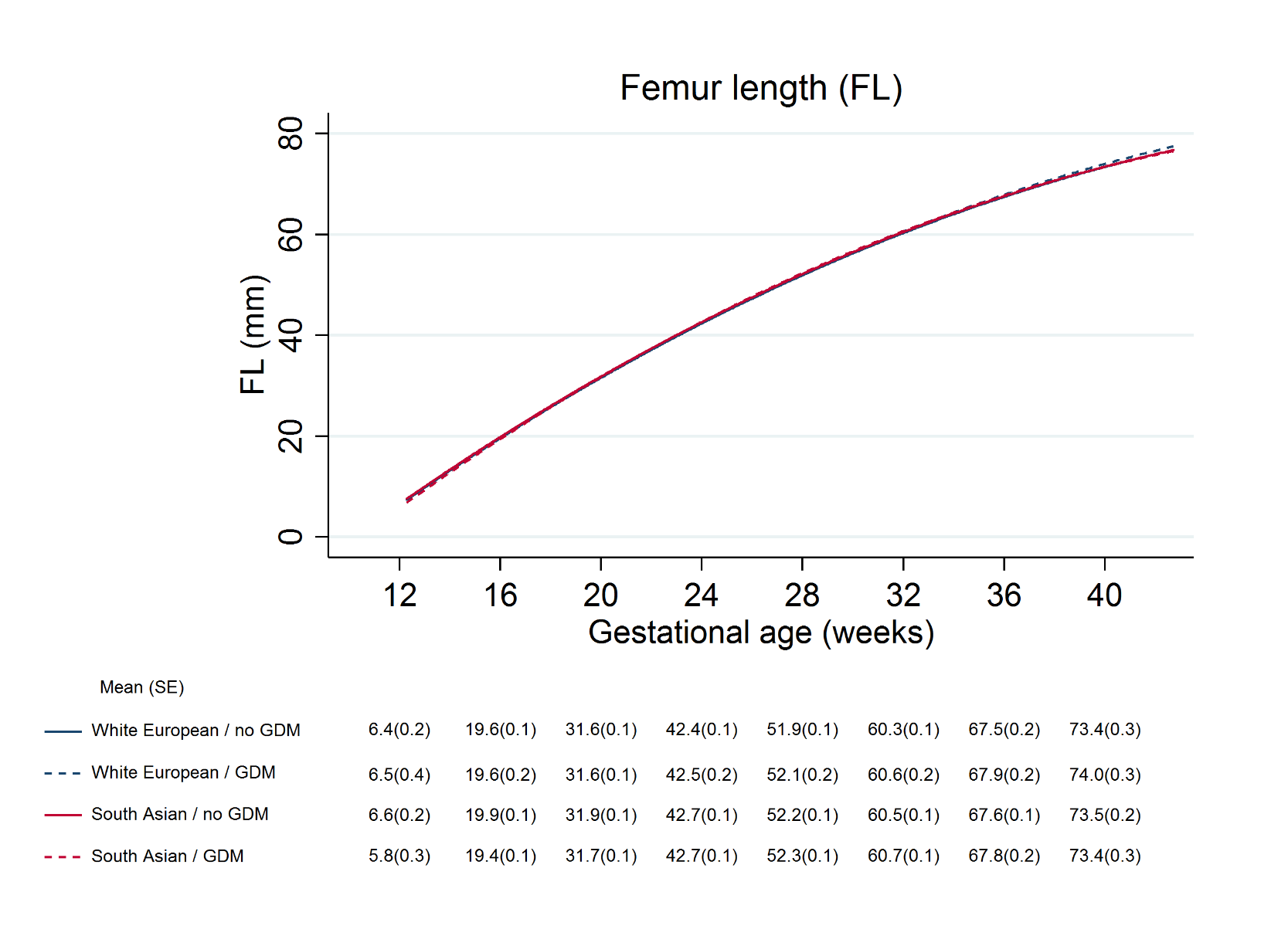
**

**C**

**
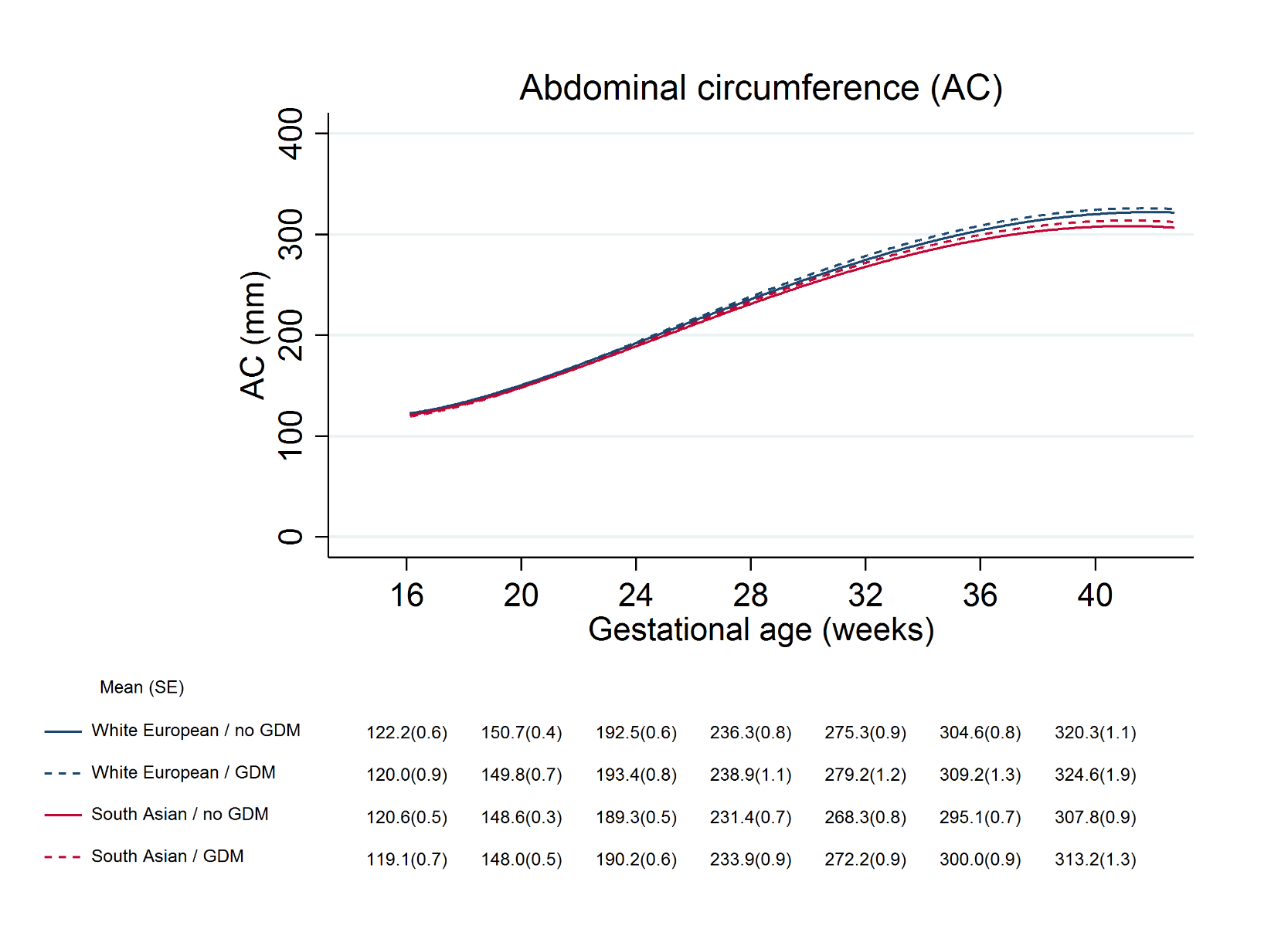
**

**D**


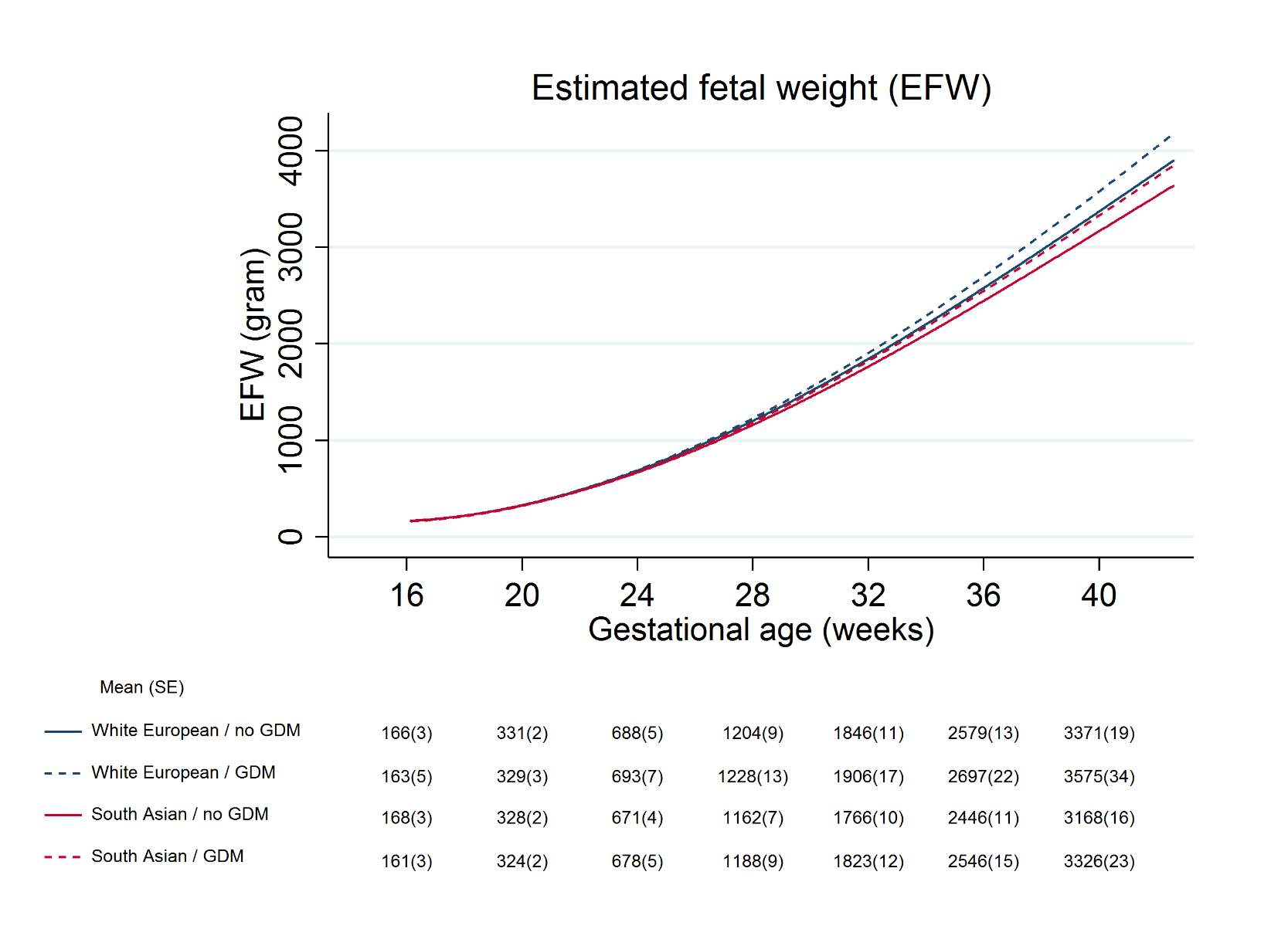


Average predicted growth trajectories of fetal head circumference (*A*), femur length (*B*), abdominal circumference (*C*) and estimated fetal weight (*D*) stratified by ethnicity (White European vs. South Asian) and gestational diabetes (yes vs. no). All growth trajectories are estimated using multilevel fractional polynomial models with adjustment for infant sex and maternal age at delivery, ethnicity parity, height, body mass index, education, smoking and alcohol use during pregnancy, and hypertensive disorders of pregnancy. In these analyses, all covariates were set to the reference category: infant sex (male), maternal age at delivery (25-29 years), parity (nulliparous), height (160-165 cm), education level (5 GSCEs), body mass index (18.5-25 kg/m^2^), smoking during pregnancy (no), alcohol during pregnancy (no), hypertensive disorders of pregnancy (no). Predicted means by ethnicity gestational diabetes are tabulated below at 4-weekly gestational age intervals from 12/16 weeks to 40 weeks. Abbrevations: GDM = gestational diabetes.
