# Supplementary material for: Gestational diabetes and ultrasound-assessed fetal growth in South Asian and White European women: findings from a prospective pregnancy cohort
Source: BMC Med. 2018 Nov 6;16:203. doi: 10.1186/s12916-018-1191-7 (PMC6219043; doi:10.1186/s12916-018-1191-7)
Supplement: Supplementary file 2 — Supplementary tables and figures including detailed information on study populations, additional data supporting our findings, and results from sensitivity analyses. (DOCX 25 kb) [file 12916_2018_1191_MOESM2_ESM.docx]

**Supplementary Methods**

***Statistical analysis of fetal growth trajectories with model specification***

Descriptive statistics of the repeat ultrasound and birth anthropometric measurements in the analysis cohort are summarized in **Table S2 in Additional file 1**.

We used multilevel models for repeat measurement data to estimate the growth trajectory of each fetal parameter [head circumference (HC), femur length (FL), abdominal circumference (AC) and estimated fetal weight (EFW)]. These multilevel models comprise of two levels: measurement occasion (level 1) clustered within the individual (level 2). This approach includes all singletons with at least 1 fetal measurement under the missing at random assumption, and accounts for the within-person correlation in repeat measures. Standard errors of multilevel models reflect both sampling variability and differences in the number of repeat measurements, such that trajectories in those with fewer repeat measurements are less precisely estimated than those with more. In a few occasions (n = 16), more than 1 fetal measurement was recorded at the ultrasound scan assessment. As all fetal measurements were entered in the multilevel models, measurement variation at ultrasound examination was accounted for in the analyses.

Fetal ultrasound and birth measurements were approximately normally distributed at different gestational ages and were entered untransformed in analyses. We used fractional polynomial curves with two powers of gestational age from a set of powers (-2, -1, -0.5, 0, 0.5, 1, 2, 3) to identify the best-fitting trajectory for each fetal growth parameter. The best fitting growth trajectory was identified by the same set of powers in both ethnic groups **(Figure S2 in Additional file 1)**. The best fitting powers for HC (powers 1 and 3), FL (powers 1 and 2), AC (powers 2 and 2) and EFW (powers 2 and 3) were entered as fixed and random effects at the individual level (level 2) of the model, allowing each individual to have its own growth trajectory. In HC, AC and EFW models, we further allowed the occasion level (level 1) residuals to vary over time to account for differences in measurement error between ultrasound and birth measurements. For HC and FL, we were able to estimate trajectories from 12 weeks to birth and for AC and EFW from 16 weeks to birth; there were too little data prior to these time points for precise estimation. Model fit was judged by comparing predicted versus observed measurements during different time periods of gestation by checking the distribution of individual level residuals. The predicted measurements of all fetal growth models were broadly consistent with those observed **(Table S5 in Additional file 1)**. The multilevel fractional polynomial model for each fetal parameter in all study participants (White Europeans and South Asians) is specified below:

Head circumference (HC):

*y*_ij_ = *β*_0_ + *µ*_0j_ + (*β*_1_ + *µ*_1j_)*t_ij_* + (*β*_2_ + *µ*_2j_)*t_ij_^3^* + *e*_0ij_ + (*e*_1ij_)*t_ij_* + (*e*_2ij_)*t_ij_^3^*

Femur length (FL):

*y*_ij_ = *β*_0_ + *µ*_0j_ + (*β*_1_ + *µ*_1j_)*t_ij_* + (*β*_2_ + *µ*_2j_)*t_ij_^2^* + *e*_ij_

Abdominal circumference (AC):

*y*_ij_ = *β*_0_ + *µ*_0j_ + (*β*_1_ + *µ*_1j_)*t_ij_^2^* + (*β*_2_ + *µ*_2j_)*t_ij_^2^*log*t_ij_* + *e*_0ij_ + (*e*_1ij_) *t_ij_^2^* + (*e*_2ij_) *t_ij_^2^*log*t_ij_*

Estimated fetal weight (EFW):

*y*_ij_ = *β*_0_ + *µ*_0j_ + (*β*_1_ + *µ*_1j_)*t_ij_^2^* + (*β*_2_ + *µ*_2j_)*t_ij_^3^* + *e*_0ij_ + (*e*_1ij_) *t_ij_^2^* + (*e*_2ij_)*t_ij_^3^*

where *y*_ij_ is the value of HC, FL, AC or EFW at gestational age (*t_ij_)* for individual *j*. Gestational age was set at 12 weeks (HC, FL) or 16 weeks (AC, EFW) with *β_0 ,_ β_1_* _and_ *β_2_* representing respectively the average intercept and coefficients for each power term of gestational age. The second power term for AC was multiplied by the log of gestational age, as we cannot enter the same power terms in a model. Deviations from the average intercept and power term coefficients for individual *j* are represented by *µ*_0j_, *µ*_1j_ and *µ*_2j_ respectively. The *e*_ij_ term describes the deviation of the *i^th^* measurement on the *j^th^* individual from the individual growth curve. This is the residual error term. For HC, AC and EFW, the residual error was allowed to vary over time (to account for differences in measurement error between ultrasound and birth measurements) by defining *e*_ij_ as a function of each power term of gestational age (*e*_1ij_ and *e*_2ij_). The increase in variance over time was largest for AC and EFW, reflecting the difficulty of AC measurement towards the end of pregnancy because of the ‘hunched’ posture of the fetus. In all models, off-diagonal parameters of the level 1 variance/covariance matrix were set to zero.

Differences in fetal growth by ethnicity and gestational diabetes (GDM)/gestational glucose were adjusted for infant sex (model 1) and additionally for potential confounding by maternal age, parity, height, BMI, education level, smoking and alcohol use during pregnancy, and hypertensive disorders of pregnancy (model 2). These covariates are likely to be confounders of the association of GDM with fetal growth but their inclusion in the multivariable model may overadjust the association of ethnicity with fetal growth. However, for joint modelling of GDM and ethnicity, we needed a consistent set of covariates. We evaluated the potential of overadjustment by comparing the multivariable adjusted estimates with estimates from the model with adjustment for infant sex only. Because estimates with adjustment for infant sex only were of similar magnitude **(Table S7 in Additional file 1)**, the potential of overadjustment appears to be minimal.

Ethnicity and covariates were included as main effect and as interactions with each of the power terms of gestational age in models estimating fetal growth trajectories in South Asians vs. White Europeans. Overall p values for the interaction of ethnicity with all fetal coefficients (at baseline and change by gestational age) were derived from these models to assess potential differences in fetal growth. The same approach was used for analysing differences in fetal growth by GDM/gestational glucose, and for joint associations of ethnicity and GDM.

Apart from evaluating overall differences in fetal growth trajectories, we also estimated differences in mean fetal HC, FL, AC and EFW at 4-weekly intervals from 12 or 16 weeks onwards for ease of clinical interpretation. Mean differences in fetal size by ethnicity and GDM/gestational glucose were reported in absolute original units (i.e. mm and grams) and also proportionally as the ratio of the observed difference to the mean at each time point, as we might expect absolute differences to increase with increasing gestational age as fetal size increases.

***Handling of missing covariate data***

In total, 2353 singleton pregnancies (22.0%) had missing data on at least one of the potential confounders, with missing data being most for maternal early pregnancy BMI (17.3%) and least for maternal parity (3.3%). Missing covariate data were imputed under the ‘missing at random assumption’ using multivariate multiple imputation with chained equations, and 10 imputed datasets were generated. The imputation models were specified for each fetal growth parameter (HC, FL, AC and EFW) and included up to 3 repeat measures of fetal size (< 20 weeks’ gestation, 20-40 weeks’ gestation and birth) with corresponding gestational ages, ethnicity, GDM and fasting and 2-hour postload glucose, all potential confounders included in any model, and additional obstetric and neonatal characteristics that were not included in any of our analyses but that were associated with some missing data (stillbirth, hypothermia and 5-min Apgar score).
